# Etv2 regulates enhancer chromatin status to initiate Shh expression in the limb bud

**DOI:** 10.1038/s41467-022-31848-6

**Published:** 2022-07-21

**Authors:** Naoko Koyano-Nakagawa, Wuming Gong, Satyabrata Das, Joshua W. M. Theisen, Tran B. Swanholm, Daniel Van Ly, Nikita Dsouza, Bhairab N. Singh, Hiroko Kawakami, Samantha Young, Katherine Q. Chen, Yasuhiko Kawakami, Daniel J. Garry

**Affiliations:** 1 Lillehei Heart Institute, University of Minnesota, Minneapolis, MN 55455 USA; 2 Stem Cell Institute, University of Minnesota, Minneapolis, MN 55455 USA; 3 Department of Genetics, Cell Biology and Development, University of Minnesota, Minneapolis, MN 55455 USA; 4 Present Address: Hamre, Schumann, Mueller & Larson, P.C., Minneapolis, MN 55402 USA; 5 Present Address: Mitchell Hamline School of Law, St. Paul, MN 55105 USA

**Keywords:** Pattern formation, Transcriptional regulatory elements, Morphogen signalling, Differentiation, Chromatin remodelling

## Abstract

*Sonic hedgehog* (*Shh*) is essential for limb development, and the mechanisms that govern the propagation and maintenance of its expression has been well studied; however, the mechanisms that govern the initiation of *Shh* expression are incomplete. Here we report that ETV2 initiates *Shh* expression by changing the chromatin status of the developmental limb enhancer, ZRS. *Etv2* expression precedes *Shh* in limb buds, and *Etv2* inactivation prevents the opening of limb chromatin, including the ZRS, resulting in an absence of *Shh* expression. *Etv2* overexpression in limb buds causes nucleosomal displacement at the ZRS, ectopic *Shh* expression, and polydactyly. Areas of nucleosome displacement coincide with ETS binding site clusters. ETV2 also functions as a transcriptional activator of ZRS and is antagonized by ETV4/5 repressors. Known human polydactyl mutations introduce novel ETV2 binding sites in the ZRS, suggesting that ETV2 dosage regulates ZRS activation. These studies identify ETV2 as a pioneer transcription factor (TF) regulating the onset of *Shh* expression, having both a chromatin regulatory role and a transcriptional activation role.

## Abstract (web summary)

The embryonic limb bud is known to be patterned by a Shh morphogen gradient, though how Shh expression is activated remains less clear. Here the authors show that Etv2 acts as a pioneer transcription factor to mediate accessibility of the ZRS enhancer and initiate Shh expression.

## Introduction

Regulation of chromatin structure constitutes a critical aspect of gene regulation. “Closed” or silent chromatin is occupied by nucleosomes and are inaccessible to most transcriptional activators, whereas “open” or active chromatin can be recognized by a cohort of transcriptional activators and is responsive to transcriptional activation cues^1^. A limited number of transcription factors (TFs), known as pioneer factors, possess the ability to open closed chromatin and allow subsequent TFs to bind the opened chromatin, initiate gene expression and specify fate decisions^2,3^. The mechanism of chromatin modification appears to vary from TF to TF^4^. Pioneer TFs, represented by FOXA1, ASCL1, SOX2, OCT3/4, and KLF4, target closed nucleosomal DNA and can induce a local opening of chromatin, establishing a permissive state for transcription^4^. Other TFs such as c-MYC, MYT1L, and SCL do not bind closed chromatin, but modify open chromatin, allowing downstream TFs to bind the DNA and activate transcription^3^. The majority of classically-defined TFs, such as those identified by reporter gene assays, do not have chromatin modification capabilities and function by binding to chromatin domains that were made available by the chromatin-modifying TFs^5^.

Limb development has been used as an experimental paradigm to decipher fundamental developmental principles that govern morphogenesis^6,7^. More recently, this system has been used to enhance our understanding of epigenetic mechanisms, changes in chromatin structure, and non-coding regulatory sequences that control cell type-specific gene expression in developing embryos^8–11^. Sonic Hedgehog (SHH) is expressed in the posterior margin of developing limb buds, called the zone of polarizing activity (ZPA), and regulates anterior-posterior patterning and proliferative expansion of chondrogenic progenitor cells in the limb^7,12,13^. Ablation of the *Shh* gene results in loss of distal-posterior skeletal elements including the posterior four digits in the mouse^14^, and ectopic expression of *Shh* in the anterior limb bud causes formation of extra anterior digits (see 12 for review). Localized expression of *Shh* in the limb bud can be divided into three phases: initiation, maintenance/expansion, and termination. During the initiation phase, *Shh* expression is induced in a limited cell population located at the posterior margin of limb buds (in mouse, previously reported to be expressed around embryonic day (E) 9.75 and E10.25 in forelimb and hindlimb buds, respectively)^15^. SHH induces nascent limb bud cells to form polarized progenitor pools as a ZPA signal^12^. *Shh* expressing cells contribute to the distal posterior skeletal elements of the limb, including the two posterior digits^16^. During limb outgrowth, the *Shh* expression domain expands in the distal posterior region as its expression level peaks at E11.5 (the maintenance phase) and extinguishes by E12.5 (the termination phase).

Limb bud-specific *Shh* expression is regulated by a 1.7 kb *cis*-regulatory module, termed the ZPA regulatory sequence (ZRS) located approximately 0.85 Mbp upstream from the *Shh* transcription start site^17–20^. Targeted deletion of the ZRS in the mouse results in a failure to express *Shh* in the limb bud and disrupts the formation of the distal-posterior skeletal elements of the limb^21^. Multiple polydactyl mutations in human and other animals have been mapped within the ZRS region, underscoring the importance of the ZRS in *Shh* expression and limb morphogenesis^17,22^.

Studies over the last two decades have identified positive and negative regulators of the ZRS. HAND2 and HOXD13 proteins directly bind to the ZRS and activate *Shh* expression^23–26^. ETS/ETV family TFs also act through the ZRS to regulate *Shh* expression at E11.5, during the maintenance/expansion phase of *Shh* expression^27,28^. These studies suggested ETS1 and GABPa as the activating members of the ZRS. Furthermore, ETV4, an ETS family repressor, was shown to negatively regulate transcription through the ZRS. Prior to *Shh* expression, fibroblast growth factor (FGF) signaling from the distal limb primes the ZRS to a poised but inactive state in cells across the distal limb mesenchyme. ETV4, a repressor ETS protein induced by FGF8, prevents *Shh* expression in the anterior limb bud by recruiting the histone deacetylase HDAC2, while GABPa, an activator ETS protein, activates *Shh* expression in the posterior limb bud by recruiting the histone acetyltransferase p300. This antagonism of TFs was proposed as a mechanism to limit *Shh* expression to the posterior limb mesenchyme^27–30^. Although these studies highlight our current understanding of *Shh* regulation, these findings are limited to the maintenance and expansion phases of *Shh* expression, and our knowledge regarding the mechanisms that initiate *Shh* expression in limb buds remains incomplete^7,12,31^.

ETV2 (ETS variant 2) is a member of the ETS/ETV family. Studies from our laboratory and others have demonstrated that ETV2 is a master regulator of the hematoendothelial lineages^32–36^. *Etv2*-mutant embryos are lethal by E9.5 due to an absence of endothelial and hematopoietic lineages. As *Etv2* mutants do not survive until the limb development stages, the role of ETV2 in limb development has not been previously described^34,37,38^. In the present study, we demonstrate that *Etv2* expression precedes that of *Shh* expression and that ETV2 and SHH are coexpressed in the developing limb bud. We provide evidence using overexpression and loss-of-function strategies to demonstrate that ETV2 binds the ZRS in vivo, relaxes the chromatin and promotes transcriptional regulation of *Shh* expression. Further, using ATAC-seq analysis, we determined that ETV2 regulates the chromatin landscape globally in the limb bud and acts as a pioneer factor during limb development. We also show that ETV2 acts as a transcriptional activator through the ZRS region and is antagonized by ETV4 and ETV5 transcriptional repressors. Our data places *Etv2* as an upstream regulator of *Shh* expression and a pioneer TF that globally regulates chromatin landscape during limb development.

## Results

### *Etv2* is expressed in the posterior region of the limb bud

**Fig. 1:**
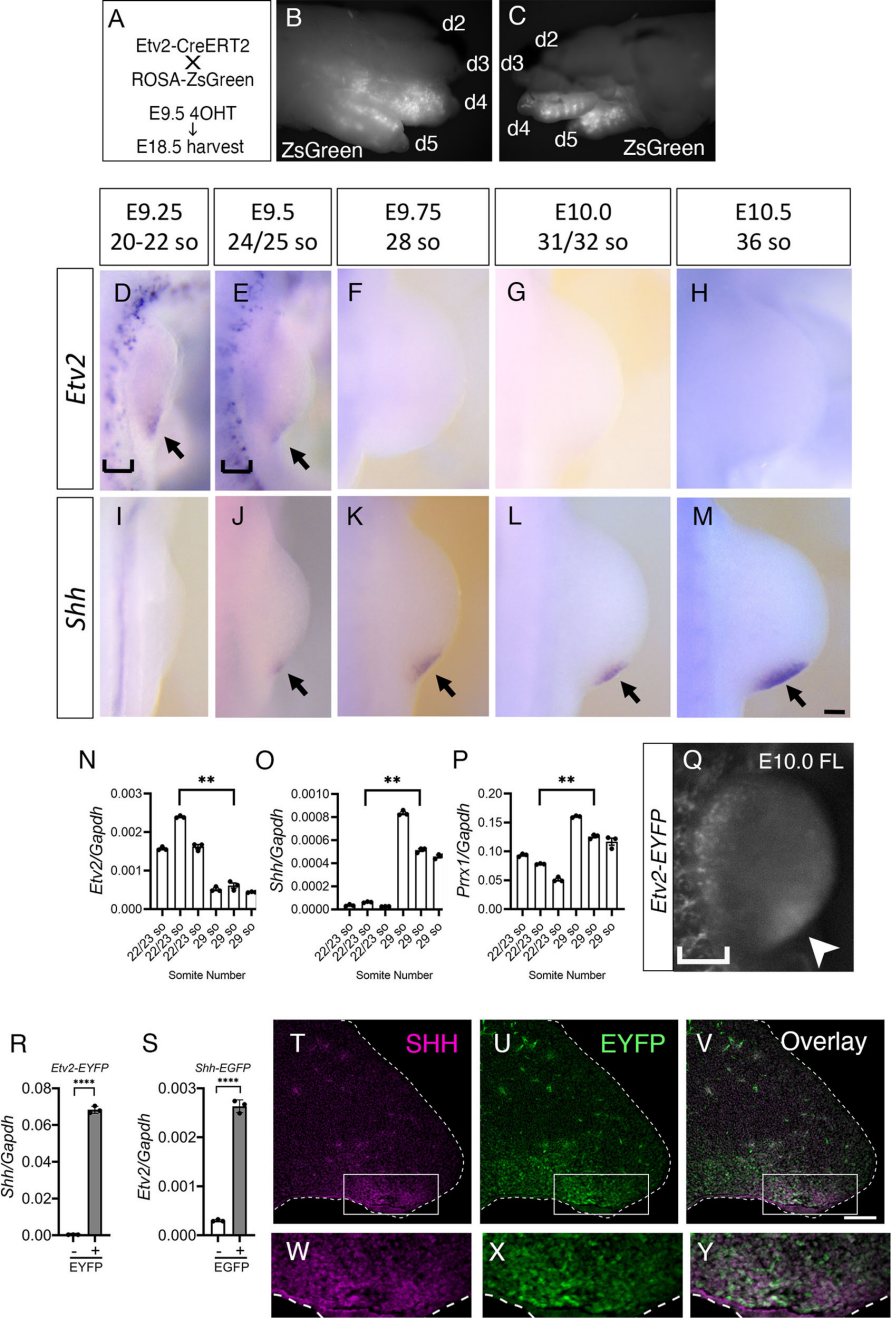
*Etv2* is expressed in the developing limb bud, preceding and overlapping with *Shh*. **A**–**C** Breeding and pulsing scheme of this analysis (A). Whole mount epifluorescence images of dorsal (**B**) and ventral (**C**) views of a forelimb are shown. d, digit. **D**–**M** In situ hybridization of *Etv2* (D-H) and *Shh* (I-M) at the embryonic stages indicated. Arrows in **D**, **E**, and arrows in **J**–**M** indicate *Etv2* and *Shh* expression in forelimb buds, respectively. Brackets in **D** and **E** indicate *Etv2* expression in the endothelial lineage. At least three embryos were analyzed at each stage for each marker. **N**–**P** qRT-PCR analyses of *Etv2* (**N**), *Shh* (**O**), and *Prrx1* (**P**) gene expression. Transcripts from forelimb buds at 22/23 somite stage (E9.25-E9.5) and 29 somite stage (E10.0), respectively from three embryos were analyzed. Sex of the embryos were not determined. Each bar represents individual embryo. Statistical test: One-way ANOVA with Tukey’s multiple comparisons test. **P-value < 0.0001 between the E9.25-E9.5 (22/23 somites) and E9.75-E10 embryos (29 somites). **Q** Whole-mount analysis of the developing forelimb of an E10.0 *Etv2-EYFP* transgenic embryo. Arrowhead points to *Etv2* promoter-driven EYFP expression in the limb bud. Bracket indicates *Etv2-EYFP* expression in the endothelial lineage. **R** *Shh* expression in *Etv2*-EYFP^+^ cells. CD31^−^Tie2^−^*Etv2*^-^EYFP^+^ (gray bar) and CD31^−^Tie2^−^*Etv2*^-^EYFP^-^ (white bar) populations were compared. *Shh* was enriched in the CD31^−^Tie2^−^*Etv2*-EYFP^+^ population^.^ *n* = 3. Statistical test: unpaired *t*-test *P-value < 0.0001 between the two groups. **S** *Etv2* expression in *Shh-*EGFP cells. CD31^−^Tie2^−^*Shh*-EGFP^+^ (gray bar) and CD31^−^Tie2^−^*Shh*-EGFP^-^ (white bar) populations were compared. *Etv2* expression was enriched in CD31^−^Tie2^−^*Shh*-EGFP^+^ population. *n* = 3. Statistical test: unpaired *t*-test. *P-value <0.0001 between the two groups. **T**–**Y** Immunohistochemistry showed overlap of SHH^+^ and EYFP^+^ cells at the posterior margin of E10.5 hindlimb buds from *Etv2-EYFP* transgenic embryos. A transverse section of hindlimb bud was stained with antibodies to SHH (**T**, **W**) and GFP, which detects EYFP (**U**, **X**). **V** and **Y** are overlays of **T** & **U** and **W** & **X**, respectively. Boxed regions of **T**–**V** are enlarged in **W**–**Y**. Broken lines mark the outline of the limb bud. At least three embryos were analyzed with similar results for histological analyses. Scale bars indicate 100 μm. Data in the graphs are presented as mean values + /− SEM. Source data are provided as a Source Data file.

Previous studies have demonstrated that *Etv2* is a critical regulator of hematoendothelial lineages^34^. To evaluate whether *Etv2* is expressed during later organogenesis of an embryo, we pursued a lineage tracing strategy. We engineered a mouse line with a tamoxifen-inducible Cre under the control of the 3.9 kb-*Etv2* promoter (*Etv2-CreERT2*) crossed to the *Rosa26-ZsGreen* reporter line (Fig. 1A). 4-hydroxy tamoxifen (4OHT) was injected at E9.5, by which stage the initial phase of yolk sac hematopoiesis and embryonic angiogenesis has ceased and *Etv2* is no longer expressed in the hemato-endothelial lineages. Embryos were analyzed at E18.5. As expected, there was no noticeable ZsGreen protein expression in the blood and vasculature. Interestingly, prominent ZsGreen signals were observed in the posterior two digits of both forelimbs and hindlimbs, which are known to be derived from the *Shh-*expressing lineage (Fig. 1B, C)^16^. To examine the cell types that ZsGreen-positive cells give rise to, digits were sectioned and immunohistochemically analyzed (Fig. S1). ZsGreen positive cells were found scattered in the posterior digits (Fig. S1A). Notably, there was no overlap between ZsGreen and endomucin- or desmin-positive cells, indicating that the labeled cells were not of endothelial or myogenic lineages (Fig. S1C–S1H).

To identify the stages when *Etv2* is expressed during limb development, we undertook a series of in situ hybridization studies. In addition to the previously-described expression in angioblasts and endothelial cells (brackets in Fig. 1D, E), we observed a distinct, transient *Etv2* expression at the posterior margin of E9.25-E9.5 forelimb buds (Fig. 1D, E, arrows). The expression was down-regulated by E9.75 (Fig. 1F). The onset of expression was earlier than that of *Shh*, which became detectable at E9.5 (Fig. 1J–M, arrows). In the hindlimb buds, *Etv2* expression became detectable at approximately E9.75 and also preceded that of *Shh* (Fig. S1I–S1L). The earlier onset of *Etv2* expression than *Shh* and the rapid decline of expression was further confirmed using qRT-PCR and RNA isolated from forelimb buds at E9.25-E9.5 (22/23 somites) and E10.0 (29 somites) embryos (Fig. 1N–P). Previously, we established that the *Etv2-EYFP* transgenic reporter, which harbors the 3.9 kb promoter of *Etv2*, recapitulates *Etv2* expression in the hemato-endothelial lineages with high sensitivity^34,39,40^. As the half-life of EYFP protein is longer (up to a few days) than the rapidly-degraded mRNAs, EYFP can be used as a short-term lineage tracer to detect the population of cells that expressed *Etv2* in a limited time frame. Close examination of the forelimb buds of these embryos revealed the presence of the EYFP signal at the posterior margin of the limb buds, confirming that the 3.9 kb hemato-endothelial enhancer/promoter of *Etv2* also regulated *Etv2* expression in the limb bud and that *Etv2*-expressing cells contributed to the posterior population of the limb buds (arrowhead in Fig. 1Q).

The lineages of *Etv2*-EYFP-labeled cells in E10.5 limb buds were analyzed by immunohistochemistry (Fig. S2, S3). The *Etv2*-EYFP signal was observed in two populations of cells: the endothelial lineage that co-expressed endomucin, which was present throughout the limb bud (Fig. S2; arrowheads in S2B, S2D, and S2F point to endothelial cells that co-express endomucin and *Etv2*-EYFP); and a non-endothelial population that was found only in the ventro-posterior region of the limb bud (indicated by a bracket in Fig. S2C; note the population of *Etv2*-EYFP^+^ cells that do not express endomucin). *Etv2*-EYFP^+^ cells did not co-express desmin (DES), a myotome marker (Fig. S3A–S3C, arrows), or PAX3, a limb myoblast marker (Fig. S3D–S3F, arrowheads), but did express PRRX1, a limb mesenchyme marker (Fig. S3G–S3I). EYFP was absent in the ectodermal layer (Fig. S2E, arrow), including the apical ectodermal ridge (Fig. S2E, open arrow). These results demonstrated that *Etv2-*EYFP was expressed in the limb mesenchyme, in addition to the previously described endothelial lineage, but not in the myogenic or ectodermal lineages.

The posteriorly localized expression pattern and the lineage contribution to the posterior two digits shown above were reminiscent of the *Shh* expression pattern. Thus, we examined whether *Etv2* and *Shh* were co-expressed in the limb buds. E10.5 limb bud cells from the *Etv2-EYFP* reporter line and the *Shh-EGFP* knockin reporter line^16^ were sorted using FACS and analyzed for the presence of *Shh* and *Etv2* transcripts, respectively by qRT-PCR. CD31 and TIE2 positive cells were sorted out to exclude the endothelial lineage (Fig. 1R, S). *Shh* transcript was present in the CD31^−^TIE2^−^*Etv2*-EYFP^+^ population but not in the CD31^−^TIE2^−^*Etv2*-EYFP^−^ population (Fig. 1R). Correspondingly, *Etv2* transcript was enriched in the *Shh*-EGFP^+^ cell population, compared with the *Shh*-EGFP^−^ population, from the limb buds of *Shh-EGFP* knockin embryos (Fig. 1S). Importantly, low level expression of *Etv2* transcripts was found in the *Shh*-EGFP^−^ population, indicating that the *Etv2*-expressing population is broader than the *Shh*-expressing population. Next, sections of E10.5 limb bud from *Etv2-EYFP* transgenic embryos were co-stained with GFP and SHH antibodies. Significant overlap of EYFP and the SHH signal was observed at the posterior limb bud margin (Fig. 1T–Y). Overall, these results are consistent with the notion that ETV2 is expressed in the population that expresses SHH in the posterior region of the developing limb bud.

### ETV2 regulates *Shh* and cell proliferation in the limb bud

**Fig. 2:**
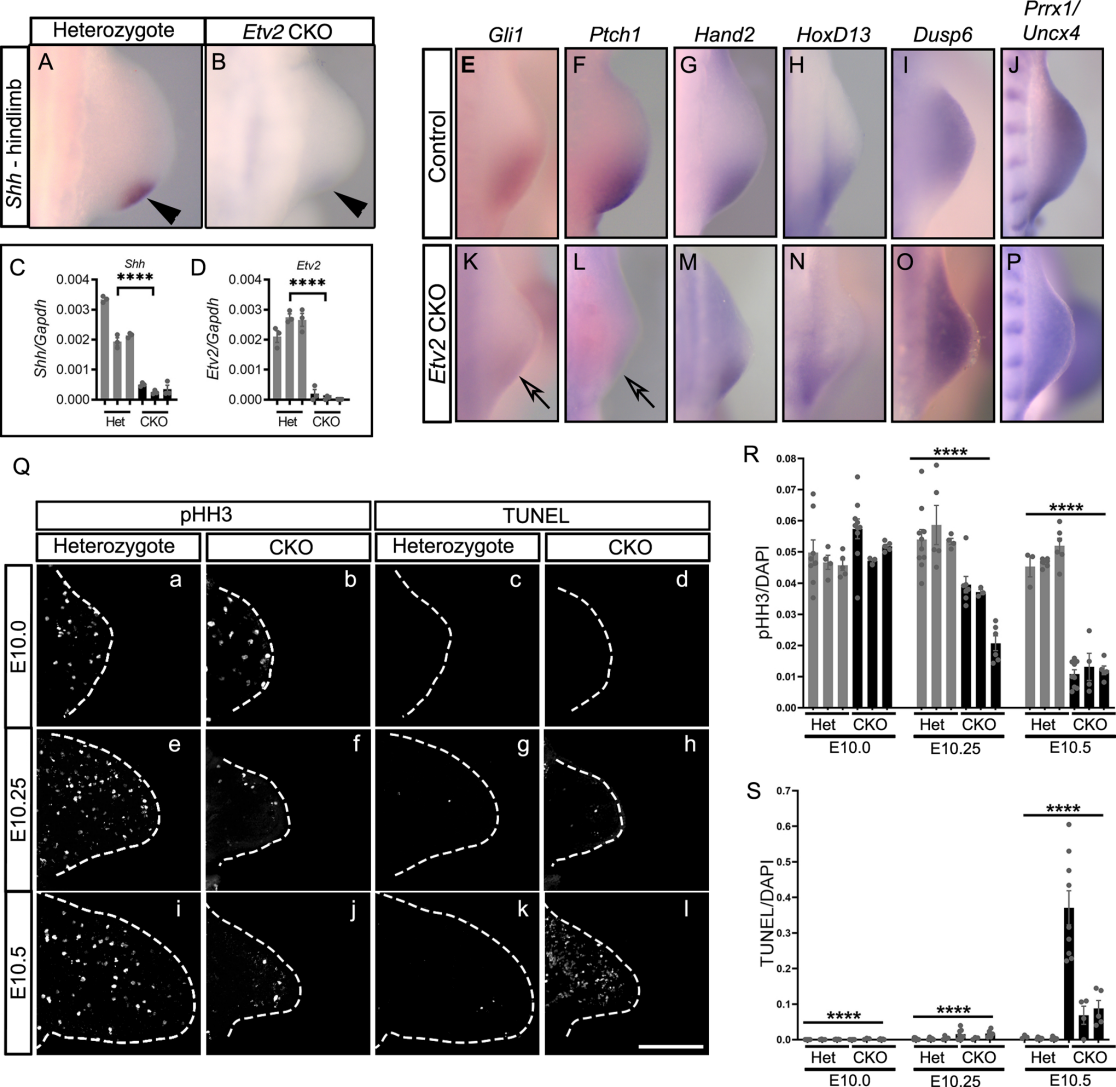
Conditional knockout of *Etv2* ablates *Shh* expression and impacts cell proliferation and apoptosis in the limb bud. Control (**A**, **E**–**J**) and *Etv2*CKO mutants (**B**, **K**–**P**) were analyzed. **A**, **B** Whole-mount in situ hybridization of *Shh* at E10.5 (36–37 somites). Arrowhead in (**A**) points to *Shh* expression, which is absent in *Etv2*CKO embryos (**B**, arrowhead). Four out of five embryos analyzed showed complete absence of *Shh* in hindlimbs. **C**, **D** qRT-PCR analysis of *Shh* (**C**) and *Etv2* (**D**) transcripts in control (gray bars) and *Etv2*CKO mutants (black bars) at E10.5 (36–37 somites). Data from three embryos are shown. Each bar represents an embryo. Statistical test: one-way ANOVA. ****P-value < 0.0001 between the Het and CKO embryos. **E**–**P** In situ hybridization analyses of indicated markers at E10.25 (32–33 somites). Note in **K** and **L** that *Gli1* and *Ptch1* expression are downregulated (arrows). At least five embryos were analyzed for **A**, **B**, **E**–**J**, with consistent results. Three embryos were analyzed for **K**–**N** and two embryos analyzed for **O** and **P** with consistent results. **Q** Immunofluorescence images of phospho-histone H3 staining (a, b, e, f, i, j), and TUNEL staining (c, d, g, h, k, l) on transverse sections of hindlimbs of heterozygotes (a, c, e, g, i, k) and *Etv2*CKO (b, d, f, h, j, i) at indicated stages are shown. Broken lines mark the border of the sections. At least three embryos were analyzed at each stage with similar results. Scale bars are 100 μm. **R**, **S** Quantification of pHH3- (**R**) and TUNEL-positive cells (**S**) in control (gray bars) and *Etv2*CKO embryos (black bars). Three embryos were sectioned and quantified. Sex of the embryos were not determined. Each bar represents an embryo. At least three sections were quantified for each bar. Statistical test: Mixed-effects model (REML) with Fixed effects (type III) Row factor analysis. *P = 0.0224, **P = 0.0062, and ****P-value < 0.0001 between the two groups. Data in the graphs are presented as mean values + /− SEM. Source data are provided as a Source Data file.

We then examined whether ETV2 regulated *Shh* expression. As *Etv2* mutant embryos were severely deformed by E9.5, we conditionally deleted *Etv2* in the lateral plate mesoderm using the *HoxB6-Cre* driver^41^. The *HoxB6-Cre* driver is active in the entire hindlimb precursor, whereas the deletion in the forelimb precursor was partial^41^. Thus, we focused our analysis on hindlimbs from *Etv2* conditional knockout (CKO) embryos. At E10.5, *Etv2*CKO and control heterozygous embryos were obtained at a Mendelian ratio (30 out of 60 embryos analyzed were *Etv2*CKO). *Etv2*CKO embryos demonstrated a severe reduction of hindlimb size and loss of *Shh* expression in the developing limb buds, while rostral development of the embryos appeared normal (Fig. 2A, B, arrowheads, S4A, S4B). While four out of five *Etv2*CKO embryos lacked *Shh* expression in the hindlimbs, all the embryos retained *Shh* expression in the forelimbs (Fig. S4C, S4D). Most *Etv2*CKO embryos were dead by E11.5, due to the late-onset angiogenic defects, consistent with a previous report^42^. qRT-PCR analysis confirmed a significant reduction of *Shh* and *Etv2* transcripts in the posterior halves of the hindlimbs of *Etv2*CKO embryos (Fig. 2C, D). In situ hybridization analysis revealed the absence of *Gli1* and *Ptch1* expression, downstream targets of the SHH signaling pathway (Fig. 2E, F, K, L, arrows). In contrast, expression patterns of *Hand2* and *Hoxd13*, known upstream activators of *Shh*, were comparable in the *Etv2*CKO and control embryos (Fig. 2G, H, M, N). FGF signaling from the ectoderm is required for proper onset of *Shh* expression^43^; however, expression of *Dusp6*, a target of FGF signaling, and *Prrx1*, a marker of limb mesenchyme, were not changed (Fig. 2I, J, O, P). Thus, the reduction of *Shh* in the *Etv2*CKO embryo was not due to the absence of upstream regulators HAND2 and HOXD13, or the lack of FGF signaling.

As limb growth was retarded in *Etv2*CKO embryos, which became noticeable as early as E10.25, we examined whether cellular proliferation or apoptosis in the developing limb bud was impacted by the lack of *Etv2*. Cross sections of hindlimb buds were stained for phospho histone H3 (pHH3) to assess proliferation, and apoptosis was examined using the TUNEL assay. The number of pHH3 positive cells started to decline in *Etv2*CKO limb buds at E10.25 and was severely reduced by E10.5 (Fig. 2Qb, Qf, Qj, R). No TUNEL-positive cells were observed at E10.0, the stage when endogenous *Shh* expression is already initiated in wildtype embryos. At E10.25, we observed a small number of TUNEL-positive cells, which became significant at E10.5 (Fig. 2Qh, Ql, S). Our observations indicated that the initial reduction of limb size was mainly due to reduced proliferation, but not due to an increase in apoptosis. Previous studies have established the role of SHH as a promoter of cell proliferation and as an anti-apoptotic factor^13^. As a loss of *Shh* is known to cause apoptosis of the limb bud mesenchyme^13^, the cell death at E10.5 is likely caused by the failure to express *Shh* (Fig. 2A, B). Given that the onset of the endogenous *Shh* expression precedes the stage of massive cell death in *Etv2* CKO embryos, our results suggest that the failure to initiate *Shh* expression is a direct consequence of an absence of *Etv2* rather than caused by loss of cells in the limb^13^.

### ETV2 relaxes ZRS chromatin and alters TF accessibility

**Fig. 3:**
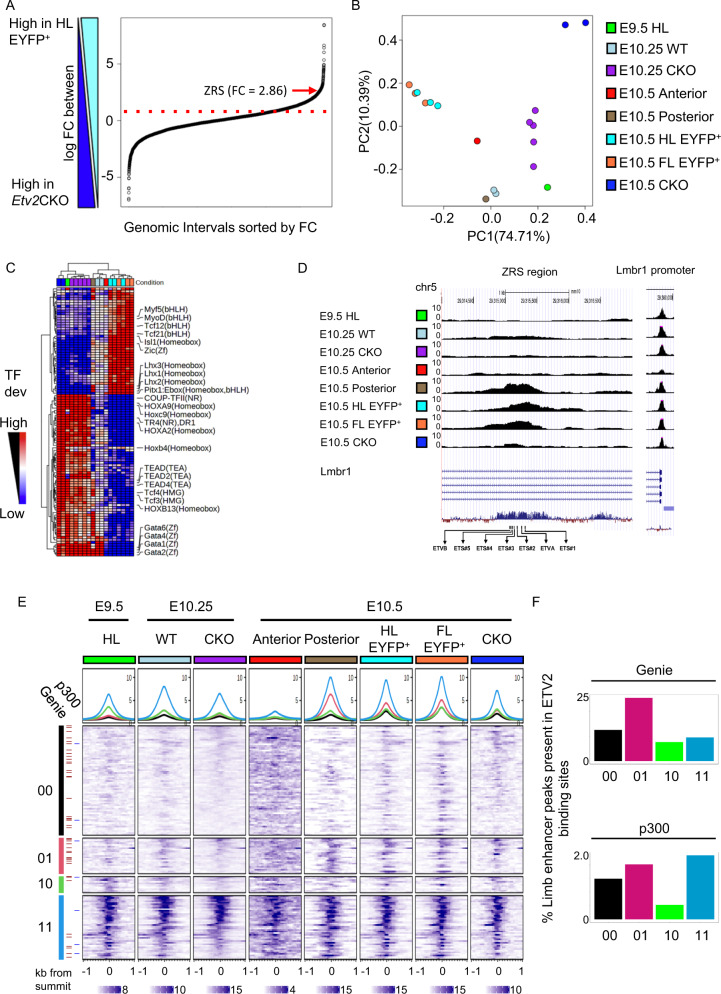
ETV2 globally affects chromatin accessibility in the limb progenitors, and the ZRS is one of the targets. **A** A plot of 5,178 conserved (>0.8) ATAC-seq intervals sorted by the fold change (FC) between HL *Etv2*-EYFP^+^ versus *Etv2*CKO samples. **B** PCA of different cell populations obtained from limb buds. Each dot represents an ATAC-seq sample (the color labels of the samples are shown in the right panel and are used throughout the figure). PC1 (explained variance: 74.71%) separated the *Etv2*-expressing (orange and teal dots) and non-expressing populations (blue, purple, and green dots) efficiently (note the separation of dots along the PC1 axis). **C** The heatmap of transcription factor (TF) deviations showed the variations of TF associated chromatin accessibility in each sample. Each row represents a TF motif, and each column represents an ATAC-seq sample. The row and column ordering were based on the clustering of the TF deviation scores derived by chromVAR. Dendrogram shown at the top reveals the global difference in TF accessibility between *Etv2-*expressing cells and non-expressing cells. **D** ATAC-seq analysis of the ZRS region (chr5: 29,314,591 – 29,316,266) (left panel) and the *Lmbr1* gene promoter region (chr5:29,377,922-29,379,008) (right panel). Locations of putative ETS and ETV binding sites are indicated at the bottom. **E** The heatmap shows the ATAC-seq density within 1 kb up- and down-stream of 7,352 putative Etv2 binding sites at different samples. The ETV2 motifs-centric regions were split into four groups according to their chromatin accessibility in E9.5 HL and E10.5 posterior, two samples that does not or does express *Etv2*, respectively. The regions are split into four groups: closed in both E9.5 HL and E10.5 Posterior (denoted as 00); closed in E9.5 HL and open in E10.5 Posterior (denoted as 01); open in E9.5 and closed in E10.5 Posterior (denoted as 10); and open in both E9.5 HL and E10.5 Posterior (denoted as 11), respectively. **F** The bar plot shows the proportion of the known limb enhancers from Limb-Enhancer Genie^45^ and the published p300 ChIP-seq peaks in each cluster, respectively. Each sample in panels **B**, **C**, **D** and **E** is represented by the same color. For wild-type samples, dissected hindlimb buds from 16 to 20 embryos were pooled for each sample. For *Etv2*CKO, samples from 3 to 5 embryos were pooled. Sex of the embryos were not determined. Each track represents average of two replicates.

Expression of *Etv2* at early stages of limb development and its known role as a master regulator of hemato-endothelial development suggests that ETV2 may also have a master regulatory role in limb morphogenesis. Thus, we examined whether ETV2 functions as an epigenetic regulator and controls gene expression during limb development. The Assay for Transposase-Accessible Chromatin using sequencing (ATAC-seq) was utilized to examine the global chromatin accessibility in limb bud cells (Fig. 3). We first compared two cell populations: one that expressed *Etv2* (CD31^−^TIE2^−^*Etv2*-EYFP^+^ cells from *Etv2-EYFP* transgenic E10.5 hindlimb) and another that lacked *Etv2* expression (unsorted E10.5 *Etv2*CKO hindlimb cells) (Fig. 3A). We identified 16,895 500-bp genomic intervals that showed a difference in ATAC-seq signals between the *Etv2*-EYFP^+^ and the *Etv2*CKO cells (DESeq2 *p*-value < 0.01 and |log2 fold change | > 1)^44^. Of these regions, 5,178 regions were evolutionarily conserved (average phastCons score > 0.8); these regions were ranked according to the fold change in ATAC-seq signal (Fig. 3A) and 873 of the loci showed more than a 2-fold difference in the ATAC-seq signals between the *Etv2*-EYFP^+^ and *Etv2*CKO cells. The ZRS region, the limb-specific enhancer of *Shh*, showed a 2.86-fold higher ATAC-seq signal in *Etv2-*EYFP^+^ cells and ranked within the top 0.3% of the conserved, differentially accessible genomic regions (red arrow in Fig. 3A). Among 110,133 200-bp ATAC-seq peaks across all samples, we found that the ZRS belonged to a cluster with 4,690 ATAC-seq peaks, with nearby genes, which were significantly associated with limb development (Fig. S5A, S5B). Thus, this initial analysis supported the notion that there is a global difference in the chromatin accessibility between *Etv2*-expressing and non-expressing cells; and the chromatin accessibility of ZRS is significantly reduced in the *Etv2*CKO hindlimb cells.

Next, we expanded the analysis of the global change of chromatin accessibility by adding more cell populations (Fig. 3B). Cells from wild-type E9.5-9.75 (25–28 somite stage) hindlimbs (E9.5 HL, prior to *Shh* expression), cells from *Shh-EGFP* E10.5 anterior hindlimbs (E10.5 Anterior, not expressing *Shh*), EGFP^+^ cells from *Shh-EGFP* E10.5 posterior hindlimbs (E10.5 Posterior, expressing *Shh*), and EYFP^+^ cells from *Etv2-EYFP* transgenic E10.5 hindlimbs and forelimbs (E10.5 HL EYFP ^+^ and E10.5 FL EYFP ^+^ , expressing *Etv2*) were collected and processed for ATAC-seq. Posterior cells from *Etv2*CKO hindlimbs at E10.25 and E10.5 (E10.25 CKO and E10.5 CKO, not expressing *Shh*) and posterior cells from E10.25 wild-type hindlimbs (E10.25 WT, expressing *Shh*) were also included. ChromVAR was utilized to investigate the chromatin accessibility of TF binding sites in the genomic intervals identified above^44^. ChromVAR used the deviation scores to represent the relative abundance of TF-associated ATAC-seq reads in each sample and the TF activity. The Principal Component Analysis (PCA) was performed on the TF deviation scores to investigate the global TF activity of each sample (Fig. 3B, C). We found that PC1, which explained the majority of the variance (74.71%), separated the *Etv2*- and *Shh-*expressing populations (*Etv2*-EYFP^+^ or *Shh-*EGFP^+^ cells from forelimb and hindlimbs) and non-expressing populations (*Etv2*CKO and E9.5 hindlimb) (Fig. 3B). These results suggested that the variance in chromatin accessibility positively correlated with the expression of *Etv2*.

Taking a closer look at 122 TFs that showed significantly different deviation scores (chromVAR variability *p-*value < 1e-5) across these samples, we found that the binding motifs of known limb development-related TFs such as LHX1/2, ISL1, and MYF5 showed higher deviation scores (indicating increased chromatin accessibility) in the *Etv2*-EYFP^+^ samples (E10.5 forelimb EYFP^+^ and E10.5 hindlimb EYFP^+^), whereas the binding motifs for HOX and GATA factors showed higher deviation scores in the *Etv2*^−^ populations (E9.5 hindlimb and *Etv2*CKO). Thus, the chromVAR analysis revealed distinct differences in the accessibility of specific TF binding sites in the presence or absence of ETV2. Notably, E9.5 hindlimb, E10.25 and E10.5 *Etv2*CKO samples shared similar TF associated chromatin accessibility patterns, suggesting that *Etv2*CKO limb bud cells maintained the chromatin structure of an early developmental stage in the absence of *Etv2* (see the clustering results shown at the top of panel C).

As ZRS was identified as a locus that has high fold-change in the ATAC-seq analysis (Fig. 3A), ATAC-seq profiles in the ZRS region of the different samples were further examined (Fig. 3D). The chromatin of the mouse ZRS region (chr5: 29314591 − 29316266) was closed (ATAC inaccessible) in the E9.5 hindlimb (E9.5 HL) and E10.5 anterior hindlimb samples (E10.5 Anterior), where *Etv2* was not expressed. In contrast, ZRS chromatin was open (accessible) in the *Shh*-EGFP^+^ cells in the posterior part of the E10.5 hindlimb (E10.5 Posterior), E10.5 hindlimb *Etv2-*EYFP^*+*^ cells (E10.5 HL EYFP^*+*^), and E10.5 forelimb *Etv2-*EYFP^*+*^ cells (E10.5 FL EYFP^*+*^), where *Etv2* was expressed (Fig. 3D, left panel). The peaks of the ATAC-seq signals overlapped with the central region of the ZRS, where ETS and ETV sequence motifs were clustered. The ZRS region in the *Etv2*CKO samples were completely inaccessible at both E10.25 and 10.5, similar to the E9.5 hindlimb samples. As a control, the promoter region of the *Lmbr1* gene, which is not under the control of the ZRS^45^ was examined. This promoter region was accessible in all samples, indicating that the lack of ATAC-seq reads in the ZRS region in E9.5 hindlimb and *Etv2*CKO samples is not due to poor sample preparation (Fig. 3D, right panel). In summary, the ATAC-seq analyses revealed a clear correlation of the relaxation of the chromatin at the ZRS with expression of ETV2.

The observation of the correlation of the chromatin relaxation and expression of *Etv2* at ZRS prompted us to investigate whether the correlation also exists at other ETV2 binding sites. We investigated 7352 putative ETV2 motifs that overlapped with 110,133 200 bp ATAC-seq peaks across all samples. The ETV2 motifs-centric regions (the genomic regions where the ETV2 motifs were at the center) were split into four groups according to their chromatin accessibility in E9.5 HL, which does not express *Etv2*, and E10.5 posterior, which expresses *Etv2*, respectively (Fig. 3E, F). We found 3,569 ETV2 motifs that are closed in both E10.5 posterior and E9.5 HL (denoted as 00), 1165 open in E10.5 posterior only (including ZRS, denoted as 01), 537 open in E9.5 HL only (denoted as 10) and 2081 open in both E10.5 posterior and E9.5 HL (denoted as 11) (Fig. 3E). Moreover, we found that the known limb enhancers from Limb-Enhancer Genie^46^ and the published p300 ChIP-seq peaks in the limb^47^ were more prevalent in the group of ETV2 motifs that were closed in the E9.5 HL and open in E10.5 posterior (group 01), compared with the group of ETV2 motifs that are closed in both conditions (group 00) (25.5% vs. 12.5% for Limb-Enhancer Genie and 2.1% vs. 1.4% for p300 ChIP-seq), suggesting that the relaxation of chromatin accessibility at limb enhancers was globally correlated with relaxation of ETV2 binding sites.

### ETV2 binds to evolutionarily conserved ETS motifs in the ZRS

**Fig. 4:**
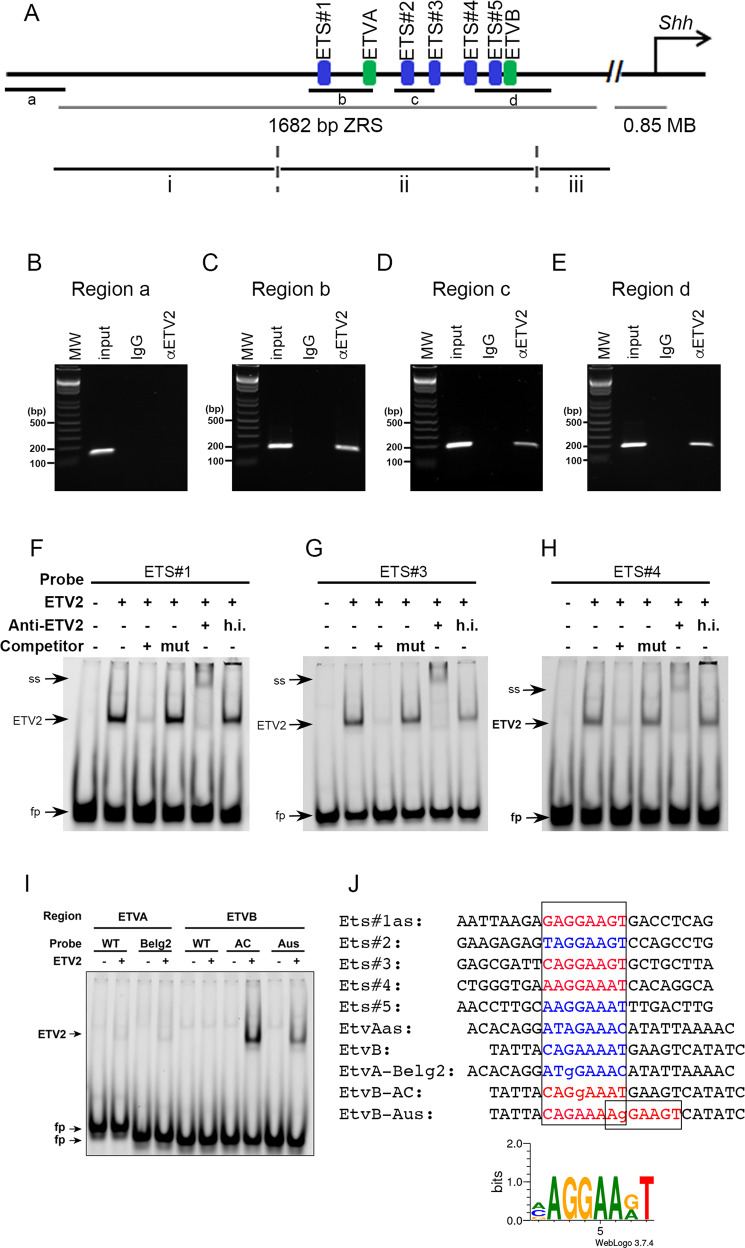
ETV2 binds to the ZRS. **A** A schematic of the ZRS region showing the ETS and ETV binding sites. Primers amplifying regions a, b, c, and d were used for ChIP assays. i, ii, and iii show the different regions of the ZRS region used for transcriptional activation assays. **B**−**E** ChIP assay with E10.5 hindlimb extracts. Hindlimbs from 41 wild-type embryos were pooled and dissociated. Cell extracts corresponding to 20 embryos were lysed and precipitated with an ETV2 antibody or the control IgG, respectively. Sex of the embryos were not determined. Immunoprecipitation with an ETV2 antibody shows binding of ETV2 to the regions b-d (**C**–**E**), but not to region a, which is located outside of the ZRS region (**B**). Input, input DNA without immunoprecipitation (positive control); IgG, Immunoprecipitation with control IgG; αETV2, immunoprecipitation with antibody against ETV2. Note that regions a-d were not amplified when pulled down with control IgG, while when pulled down with αETV2, regions b, c, and d were amplified. ChIP was repeated four times with pooled hindlimb buds from 10 to 20 embryos each with essentially the same results. **F**–**H** EMSA using three different probes representing Ets#1, #3 and #4 binding sites each show strong binding of ETV2 protein. Note that each of the specific band in **F**–**H** is competed by a corresponding wild-type competitor (3rd lane from the left, respectively) but not by a competitor with respective binding site mutated (4th lane). Specificity is further confirmed by supershifting by ETV2 antibody (5th lane), but not by a heat-inactivated antibody (6th lane, h.i.). **I** EMSA using ETVA and ETVB probes. No binding was observed with the wild-type sequences (WT). The AC and Aus mutations of the ETVB sequence bound ETV2, while the Belg2 mutation of ETVA sequence did not. **J** Alignment of probes used for EMSA. Red letters indicate the sequences bound by ETV2 and blue letters indicate sequences that were not. Alignment of the sequences recognized by ETV2 is shown by the graphical representation at the bottom. Each experiment was repeated at least three times with similar results. Source data are provided as a Source Data file.

The ATAC-seq analysis above suggested that ETV2 binds to chromatin globally and regulates the chromatin accessibility of genes expressed in limb buds. The analysis further suggested that ZRS is one of the loci targeted by ETV2. Thus, we examined whether ETV2 binds to the ZRS in vivo by chromatin immunoprecipitation (ChIP) assay. Within the ZRS region, five putative ETS binding sites having a core sequence of AGGAA(G/A)T and two putative ETV binding sites having a core sequence of AGAAA have previously been defined as ETS#1-5, ETVA and ETVB (Fig. 4A; see Fig. 4J for the sequences)^27^. Primer sets that amplify the regions outside of the ZRS (region a) and inside the ZRS (regions b-d) were used for PCR (Fig. 4A). ETV2 binding was detected at regions b-d, but not at region a (Fig. 4B–E). These experiments demonstrated that ETV2 could bind to the ZRS region encompassing multiple ETS binding sequences in the developing limb bud. EMSA was performed to compare the in vitro binding affinity to the putative ETS and ETV binding sequences. EMSA using probes that contained one of the five ETS and two ETV sites showed that ETV2 bound to ETS#1, ETS#3, and ETS#4, as the ETV2-DNA complex was formed, competed and supershifted using an ETV2 antibody (Fig. 4F–H). ETV2 did not bind to the two putative ETV binding sites, ETVA and ETVB (Fig. 4I). These studies demonstrated that ETV2 has different binding affinities to each of the ETS and ETV binding sites and binds to ETS#1, ETS#3, and ETS#4 sites in vitro.

### ETV2 alters the ZRS chromatin structure and regulates *Shh*

**Fig. 5:**
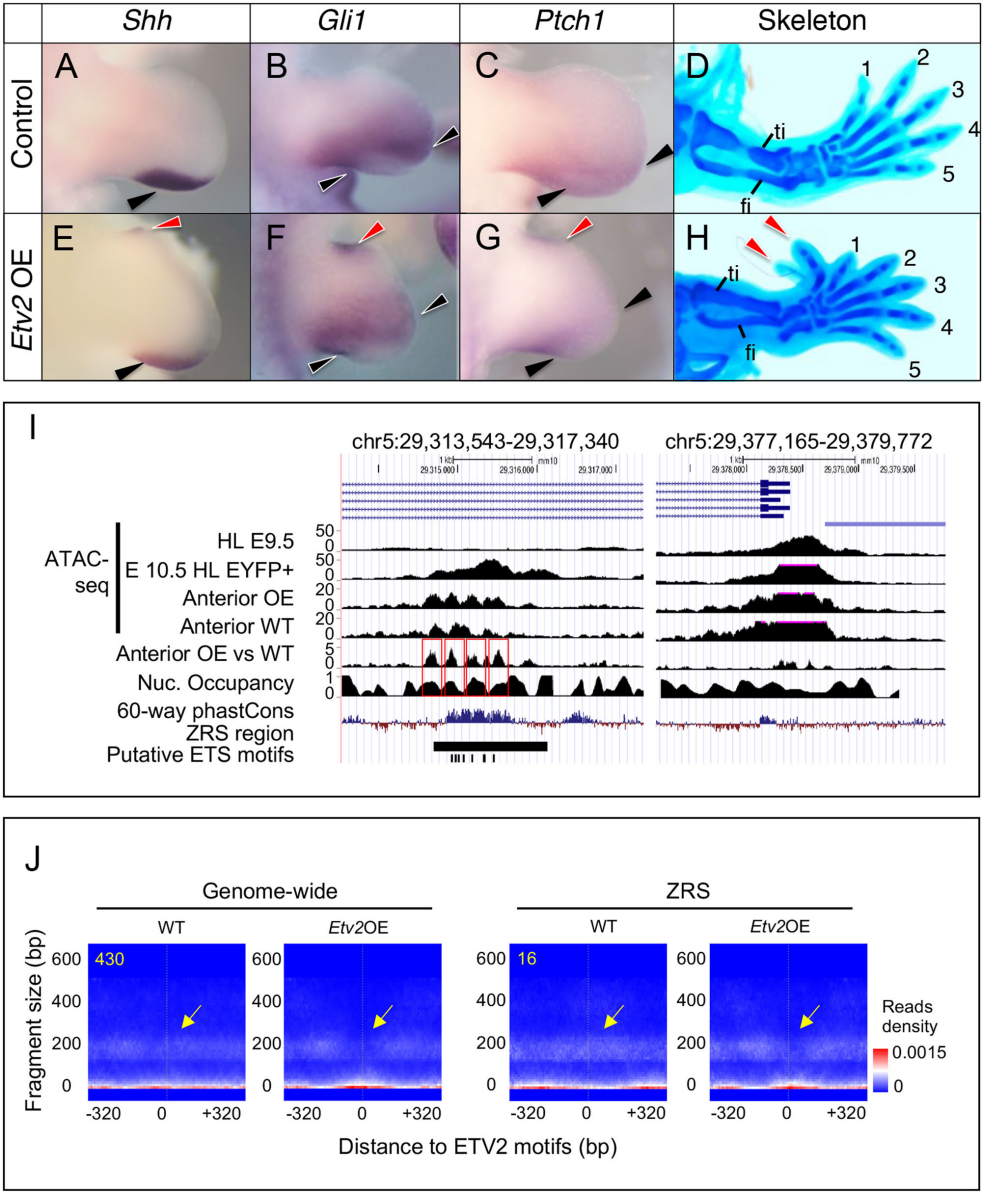
Overexpressed ETV2 displaces chromatin of limb enhancers, ectopically activates SHH signaling, and induces polydactyly. **A**–**H** Overexpression of *Etv2* resulted in ectopic induction of SHH signaling in the anterior domain and formation of extra digits. Control embryos [*HoxB6-Cre*^+/+^; *iHA-Etv2*^*Tg/+*^; *ROSA26-STOP-rtTA-IRES-EGFP*^*Tg/+*^] (**A**–**C**) and *Etv2*-overexpressors [*HoxB6-Cre*^*Tg/+*^; *iHA-Etv2*^*Tg/+*^; *ROSA26-STOP-rtTA-IRES-EGFP*^*Tg/+*^] (**E**–**G**) were analyzed at E11.5 for expression of *Shh* (**A**, **E**), *Gli1* (**B**, **F**), and *Ptch1* (**C**, **G**). Skeletal patterns of E15.5 embryos were analyzed by Alcian blue staining (**D**, **H**). Black arrowheads in **A**–**C** and **E**–**G** indicate normal expression domains, and red arrowheads indicate ectopic induction in anterior domains (**E**–**G**). Red arrowheads in H indicate ectopic digits formed anteriorly. The phenotypes were observed in hindlimbs in the following frequencies: *Shh*: two out of four embryos; *Gli1*: two out of three embryos; *Ptch1*: two out of three embryos; bilateral polydactyly: two out of three embryos. No ectopic induction or polydactyly was observed in forelimbs. **I** Overexpression of *Etv2* resulted in depletion of nucleosomes at the ZRS. For the Anterior OE and WT tracks, hindlimbs from three embryos were analyzed by ATAC-seq separately and averaged. HL E9.5 and E10.5 HL EYFP^+^ tracks show the ATAC-seq signals of E9.5 hindlimb and *Etv2*-EYFP^+^ samples from E10.5 HL, respectively. The Anterior OE and Anterior WT tracks are ATAC-seq tracks excluding the nucleosome free reads (fragment size > 150) of the anterior *Etv2*OE samples and the WT anterior samples. The Anterior OE vs WT track shows the ratio of the ATAC-seq signals of the anterior *Etv2* OE samples and the WT anterior samples. The Nuc. Occupancy track shows the nucleosome positions in the WT anterior samples. **J** The aggregated V-plot of 430 ETV2 motif-centric regions and 16 ETV2 motifs present in the ZRS region in the ATAC-seq data were significantly different (Adjusted *p*-value < 0.05) between Anterior WT and Anterior *Etv2*OE samples. The aggregated V-plots shows an increase of nucleosome free reads in the Anterior *Etv2*OE samples as compared to the Anterior WT samples. A chi-squared test with K degree of freedom (where K is the size of the latent variables) was used to compute a *p*-value of the difference between two V-plots. The Benjamini-Hochberg Procedure was used to adjust the *p*-value for multiple comparisons. The heatmap color indicates the estimated read density for estimated read counts (right). Yellow arrows point to the genomic regions changing from nucleosome-occupied to nucleosome-free from WT to Etv2 OE samples. Sex of the embryos were not determined in these experiments.

Next, we addressed whether ETV2 could alter the chromatin accessibility in the limb buds by overexpression in vivo. Since *Etv2* affects differentiation of the mesodermal lineages, global overexpression of *Etv2* would yield compound results. Therefore, we conducted targeted inducible overexpression of *Etv2* in the limb buds during the early stages of limb bud development (see Methods for the breeding scheme). Using this system, *Etv2* was induced in the *HoxB6* lineage by two injections of doxycycline at E9.5 and E10.5. Whole-mount analysis of E11.5 *Etv2*OE embryos (*HoxB6-Cre*^*Tg/+*^*; iHA-Etv2*^*Tg/+*^; *ROSA26-STOP-rtTA-IRES-EGFP*^*Tg/+*^) showed expression of EGFP in areas consistent with the reported *HoxB6-Cre* activity^41^ (Fig. S6A). Immunohistochemistry of the hindlimb sections using an anti-HA antibody confirmed overexpression of HA-ETV2 throughout the hindlimb bud (Fig. S6B). Although the overexpressed HA-ETV2 was widely distributed in the limb buds, the expression was not in all cells, likely reflecting the efficiency of the Cre-mediated recombination and the induction of ETV2 by doxycycline. Nevertheless, SHH was ectopically expressed in the anterior margin of the limb bud, in addition to the endogenous posterior expression domain (Fig. S6C). In situ hybridization showed that *Shh* as well as *Gli1* and *Ptch1*, downstream targets of SHH signaling, were ectopically expressed in the anterior margin of the limb bud (Fig. 5A–C, E–G, red arrowheads point to ectopic expression). Such ectopic expression was observed in six out of ten triple-transgenic embryos examined. At E15.5 *Etv2*OE embryos developed extra digits anteriorly, causing preaxial polydactyly (Fig. 5H, S5G, red arrowheads).

We next addressed whether chromatin accessibility was altered by overexpression of ETV2 using ATAC-seq. To conduct this analysis, nucleosome positions were first identified from the WT anterior and *Etv2OE* samples by using NucleotATAC^48^. The results revealed regularly spaced nucleosomes at the ZRS in the WT anterior sample (Fig. 5I, the Nuc. Occupancy track). Next, relaxed regions (ATAC-seq summits) in *Etv2*OE samples and WT anterior samples were identified by MACS2^49^. Calculation of the ratio of accessibility between *Etv2*OE and control embryos revealed a localized increase in chromatin accessibility at the ZRS region when *Etv2* is overexpressed (Fig. 5I, left panel, the Anterior OE vs. WT track). Notably, the genomic regions with increased chromatin accessibility correlated with the positions of nucleosomes, indicating that nucleosomes positioned at the ZRS were displaced from this region upon induction of *Etv2* (indicated by red boxes). The nucleosomes outside of the ZRS were not displaced, indicating that the nucleosome-displacing effect was specific to the ZRS (see the non-boxed region in the Anterior OE vs. WT track). Furthermore, the *Lmbr1* gene promoter, which was examined as a control, did not show displacement of chromatin (Fig. 5I, right panel, see the Anterior OE vs. WT track).

The results of ATAC-seq analysis were verified by quantitative PCR using the primers corresponding to regions a-d indicated in Fig. 4A. DNA from ATAC-seq libraries were amplified by qPCR and normalized to the amplification level from the GAPDH promoter region. Congruent with the ATAC-seq results, no amplification was found from region a, regardless of the presence or absence of *Etv2* (Fig. S6J), and the accessibility of regions b-d increased after induction of *Etv2* in both anterior and posterior halves of the hindlimbs (Fig. S6I–S6K, compare WT and *Etv2*OE samples). In conclusion, these results demonstrated specific increase of chromatin accessibility at the ZRS when ETV2 is overexpressed.

We used our recently developed tool, SeATAC (https://github.com/gongx030/seatac)^50^, to compare the 640-bp-width V-plots of 41,656 ETV2 motif-centric regions between WT and *Etv2*OE samples and identified 430 regions that had significantly increased chromatin accessibility from WT to *Etv2*OE over the ETV2 motifs (SeATAC adjusted p-value <0.05 and log ratio < −0.2)^48^. The V-plot is a dot-plot showing how sequencing reads with different fragment sizes distribute surrounding one or a set of genomic region(s). The SeATAC uses a VAE (variational autoencoder) to learn the latent representation of ATAC-seq V-plots using a CNN (convolutional neural networks) based encoder and a devolution decoder. The aggregated V-plots of these 430 regions showed an increased chromatin accessibility of ETV2 centric regions in *Etv2*OE compared with WT (Fig. 5J). ZRS ranked as the top 3.6% (191 of 5,179 regions) of the ETV2 centric regions with increased chromatin accessibility in the *Etv2*OE sample (Fig. S6L, S6M). These analyses demonstrated that ETV2 had an ability to globally target nucleosome-bound areas and relax the nucleosomes, and ZRS was among the specifically targeted areas by ETV2.

### ETV2 activates transcription through the ZRS

**Fig. 6:**
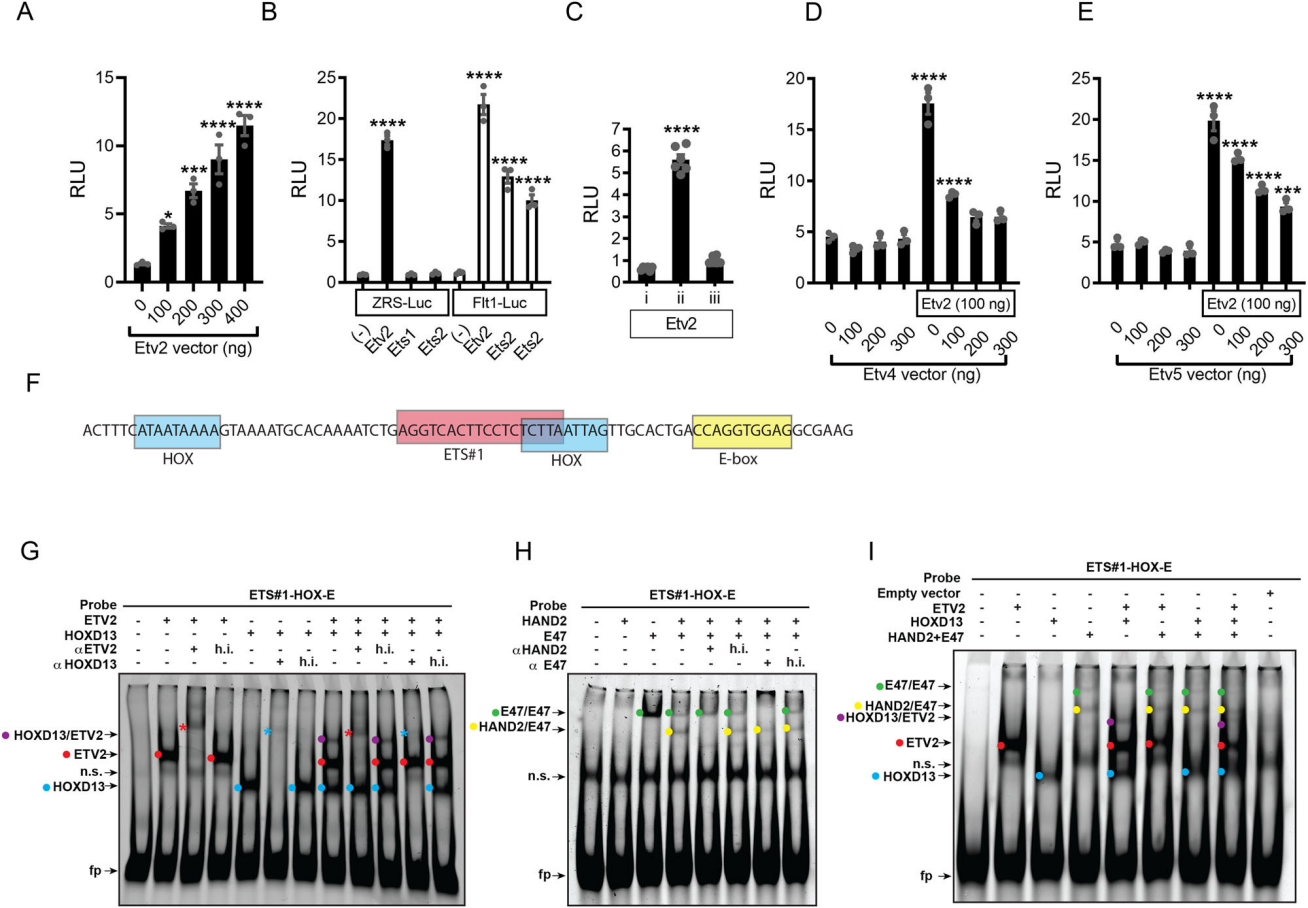
ETV2 activates transcription through the ZRS region and antagonizes ETV4 and ETV5. **A**–**E** ETV2 binds to and transcriptionally activates the ZRS region. **A** Luciferase reporter assay shows a dose-dependent activation of the ZRS in response to ETV2. *P-value of 0.0331, ***P-value 0.0004 and ****P-value < 0.0001 compared to the 0 ng Etv2. **B** The activation of ZRS-Luciferase is dependent on ETV2 but not ETS1 or ETS2 (black bars). *Flt1* promoter-luciferase reporter was used as a positive control (white bars). ****P-value < 0.0001 compared to the (-). **C** Deletional constructs of the ZRS-luciferase reporter show that the ETV2 response is limited to region ii containing the ETS-binding motifs. ****P-value < 0.0001 for ii vs i and ii vs iii. **D** Titration with Etv4 expression vector shows antagonism of ETV2 activity by ETV4. ****P-value < 0.0001 compared to the 0 ng control. **E** Titration of Etv5 expression vector shows antagonism of ETV2 transcriptional activity by ETV5. Each bar represents transfections done in quadruplicate. ****P-value < 0.0001 compared to the 0 ng Etv5 vector only control. Statistical tests: **A**, **B**, **D**, **E**, one-way ANOVA with Dunnett’s multiple comparisons test; **C**, one-way ANOVA with Tukey’s multiple comparisons test. Each experiment was repeated at least three times with essentially the same results. Representative results are shown. Data in the graphs are presented as mean values + /− SEM. **F**–**I** EMSA using HOXD13, HAND2 and ETV2 reveal that these factors bind DNA independently of each other. **F** Diagram of the oligonucleotide (ETS#1-HOX-E) used for this study containing putative HOX, ETS and E-box binding sequences. **G**, **H** EMSA of ETV2, HOXD13 and HAND2/E47 reveal that each factor binds to the probe (ns refers to nonspecific bands; fp refers to free probe). **G** shows EMSA with ETV2 and HOXD13. **H** shows EMSA with HAND2 with a universal dimerization partner of basic helix-loop-helix proteins, E47. Note that ETV2, HOXD13 and HAND2/E47 each form a DNA-protein complex (indicated by red and blue dots in **G**, and yellow dots in **H**, respectively). HAND2 does not bind to DNA by itself, but does bind when complexed with E47 (**H**, yellow dots). In this reaction, a band corresponding to an E47 homodimer was also observed (**H**, green dots). The shifted bands of ETV2 and HOXD13 are supershifted by antibodies to respective proteins (red and blue asterisks in **G**), and the shift of HAND2 and E47 is blocked by the HAND2 antibody (**H**). h.i. indicates respective heat-inactivated antibodies used as controls. The band of E47 is blocked by an antibody to E47. Purple dots indicate bands containing both HOXD13 and ETV2 (**G**). **I** EMSA with combinations of ETV2, HOXD13 and HAND2/E47 show that DNA binding activity of one protein is not affected by the presence of additional factors. Note that shift originating from ETV2, HOXD13 and HAND/E47 separately (red, blue, and yellow dots) are unaffected by the presence of additional transcription factors. Each experiment was repeated at least three times with essentially the same results. Source data are provided as a Source Data file.

The results above indicated that ETV2 had a nucleosome-displacing activity and regulates *Shh* expression at least in part by modifying chromatin accessibility. As ETV2 is known to be a transcriptional activator in the hemato-endothelial system, we examined whether ETV2 also functioned as a transcriptional activator in the regulation of *Shh*. The ZRS-Luciferase reporter that harbors the entire 1.7 kb sequence of the ZRS was used for this analysis^23^. Transfection and luciferase assays demonstrated that ETV2 activated the ZRS-Luciferase reporter in a dose dependent fashion (Fig. 6A). Among ETV2, ETS1 and ETS2, only ETV2 activated the ZRS-Luciferase reporter significantly. In a control experiment, ETV2, ETS1, and ETS2 were all able to activate the *Flt1* promoter, indicating that the activation of ZRS by ETV2 was specific (Fig. 6B). Deletional analysis of the ZRS sequence revealed that ETV2 transactivated the reporter through the central region (region ii, Fig. 4A), which harbors multiple ETS and ETV consensus sequences (Fig. 6C).

We next tested the interplay of ETV2 with ETV4 and ETV5, known repressors of *Shh*. It has been reported that blockage of ETV activity resulted in ectopic anterior expansion *Shh* and has been suggested that ETV4 and ETV5 may be antagonizing the function of unknown TF(s) that activate *Shh*^30^. Using this reporter assay system, we tested whether ETV2 can be the postulated activating factor, which is antagonized by ETV4 and ETV5. ETV4 and ETV5 did not activate or suppress the ZRS by themselves but were able to suppress the ETV2-dependent reporter activation in a dose dependent fashion (Fig. 6D, E). These results supported the model that posterior ETV2 activity and anterior ETV4 and ETV5 activity antagonize each other to regulate *Shh* expression.

Given the ability of ETV2 to activate transcription through the ZRS, we hypothesized that in polydactyly mutations, the binding of ETV2 to the ZRS might be enhanced. We chose three mutations found in familial preaxial polydactyly that altered the ETV consensus sequences and examined ETV2 binding^22,27^. ETV2 did not bind to the wild-type ETVA and ETVB sequences (Fig. 4I, ETVA-WT and ETVB-WT). Interestingly, AC and Aus mutations of ETVB changed the ETVB sequence into robust binding sequences for ETV2. Belg2 mutation of the ETVA sequence did not change ETV2 binding activity. These results demonstrated that at least in some polydactyly mutations, the binding of ETV2 to the ZRS sequence was enhanced. The sequences that ETV2 bound and did not bind are shown in red and blue, respectively, in Fig. 4J. The sequence pileup analysis of sequences bound by ETV2 revealed a preferred binding sequence of (A/C/G)AGGAA(G/A)T, which further defines the previously-defined consensus sequence of (G/C)(C/G)(C/a)GGA(T/A)(G/a)(T/c)C (two uppercase letters at a position indicate approximately equal selection, and a lowercase letter indicates a less-favored nucleotide)^51^.

### ETV2 binds to ZRS independently of HAND2 and HOXD13

Previous studies have demonstrated that HAND2 and HOXD13 bind to the ZRS in E11.0 limb bud extracts in vivo and activated *Shh* expression in vitro^23^. Thus, we tested the hypothesis that ETV2 interacts with other pioneer factors to activate the ZRS. For example, ETV2 could cooperatively bind to DNA by forming a transcription complex or cooperatively modify chromatin structure by forming a pioneer factor complex. To obtain insights to these possibilities, we used EMSA to examine whether ETV2 formed a complex with HAND2 and HOXD13 in vitro. Of the three ETS sites ETV2 binds (Fig. 4F, G, H), ETS#1 site has a potential binding sequence for E-proteins and two potential binding sites for HOX proteins within a distance of 20 base pairs (Fig. 6F). Thus, we synthesized an oligonucleotide probe encompassing these binding sites and interrogated whether cooperative binding could be detected. ETV2 alone, HOXD13 alone, or HAND2 and its binding partner E47 formed protein-DNA complexes with distinct mobilities (Fig. 6G and H, red, blue, and yellow dots, respectively). Specificity of the shifted bands were confirmed by supershifting with antibodies to ETV2 and HOXD13 (Fig. 6G, red and blue stars, respectively) or disappearance of the HAND2/E47 complex by an antibody to HAND2 (Fig. 6H). Mixing combinations of two or three TFs produced shifted bands that corresponded to each of the mixed proteins, however no new band indicative of protein-protein interaction was observed (Fig. 6I). In the combination of HOXD13 and ETV2, a faint band that was competed by both HOXD13 and ETV2 antibodies was observed (Fig. 6G and I, purple dots). This band likely reflected both proteins binding to the same probe, but not cooperatively, as the intensity of the main protein-DNA complexes (red and blue dots) did not change significantly. In summary, in the in vitro binding system (EMSA), we did not observe any indication of a direct protein-protein interaction between ETV2 and HOXD13 or ETV2 and HAND2 proteins.

## Discussion

Regulation of chromatin structure plays a gate-keeping role in development^2–4^. Closed chromatin prevents aberrant expression of genes, while open chromatin is permissive for the binding of TFs and will allow expression of the cell-type specific genes. Here, we report three important discoveries regarding the regulation of chromatin accessibility in the limb buds and define ETV2 as a critical regulatory factor of chromatin accessibility and expression of *Shh*.

Our first discovery defined a mechanism of ETV2 function as a pioneer factor to regulate the chromatin landscape in limb development. We demonstrated by multiple genetic and biochemical assays that ETV2 recognizes and relaxes the closed chromatin structures globally and promotes the downstream limb development program. Using ZRS as a representative ETV2 target, we demonstrated that chromatin relaxation correlated with the ETV2 dosage. In normal development, the chromatin of ZRS was completely closed at E9.5 but became widely accessible in the cells from posterior domain of the hindlimb at E10.5. The anterior portion remained closed at E10.5. The switching of ZRS chromatin state in the posterior domain occurred around E10.25, which showed intermediate accessibility between E9.5 and E10.5 posterior hindlimbs. In the *Etv2*CKO limbs, the ZRS remained closed at E10.25 and E10.5, and these samples clustered close to the E9.5 hindlimbs in the PCA. Close clustering of the wild-type E9.5 sample and *Etv2*CKO samples in the principal components indicates that the global chromatin accessibility of *Etv2*CKO limbs is similar to that of wild-type E9.5 limbs. Moreover, the transition of chromatin accessibility from E9.5 to E10.25 and E10.5 posterior tissue in wild-type hindlimbs supports our notion that ETV2 regulates global chromatin accessibility in the developing limb and transition of pre-patterning to *Shh*-dependent patterning phase. As apoptosis has not yet significantly increased at E10.25, it is unlikely that the observed closed chromatin structure in *Etv2*CKO limbs is due to the complete loss of ZPA cells. Instead, the TUNEL analysis supports our notion that the closed chromatin status is a direct consequence of the loss of *Etv2* function. In a gain-of-function experiment, induced overexpression of *Etv2* in limb buds enhanced chromatin accessibility. The sequences in ZRS, where the accessibility was increased by ETV2 as identified by NucleoATAC, coincided with the nucleosome positions. These data suggest that the increase in accessibility was caused by displacement of the nucleosomes. Furthermore, these areas coincided with the ETS/ETV binding sequences in the ZRS, to which ETV2 was shown to bind by in vitro (EMSA) and in vivo (ChIP) assays. The V-plot analysis of ETV2 motif centric regions in WT and *Etv2*OE samples showed that inducing *Etv2* increased the chromatin accessibility at the ETV2 sites, which is consistent with the notion that the effect of ETV2 is not limited to ZRS but is global.

These results strongly suggested the role of ETV2 as a global chromatin regulator in limb development, ZRS being one of the targets. Open chromatin was associated with ETV2 expression and loss-of-function of ETV2 resulted in closed chromatin, while overexpression of ETV2 enhanced accessibility. Collectively, these results supported our hypothesis that ETV2 functions as a pioneer factor at the ZRS.

Whether ETV2 can open the chromatin from a completely inaccessible state remains to be investigated. Recent analysis of histone modifications at the ZRS showed that FGF signaling in the distal limb bud primed the ZRS, maintaining a poised, but inactive state broadly across the distal limb mesenchyme^28^. ETV2 may require such a poised state to modify the chromatin landscape at the ZRS. We also recognize the possibility that other factors may also have an epigenetic role in the regulation of the ZRS and *Shh* expression in the developing limb and it will be further examined in future experiments. HOXD13, which was shown to have a pioneer function is one of these candidates^52^. Our in vitro studies did not support that ETV2 physically interacts with HOXD13 or HAND2. The interrelationship of ETV2 with other TFs that have pioneer function or transcriptional activation function warrants future research. ETV2’s role in other mesodermal lineages (such as hematopoietic and endothelial lineages) is intriguing in light of its ability to alter chromatin accessibility. It has been shown that ETV2 alone, or in combination with other factors is able to reprogram fibroblasts, skeletal muscle, and amniotic cells to the endothelial lineage^35,53–57^. Such master regulatory role suggests that ETV2 may be able to relax completely closed chromatins at the relevant target loci.

Our second discovery defined ETV2 as the TF of the ETS/ETV family members that is essential for initiation of *Shh* expression. In addition to the chromatin-regulatory activity, ETV2 was able to bind to ZRS and activate transcription through ZRS in a reporter gene assay. The activity of ETV2 was antagonized by negative TFs ETV4 and ETV5. *Etv2* was expressed in the developing limb buds preceding *Shh* and was co-expressed with *Shh* in the posterior domain of the limb bud. Conditional knockout of *Etv2* in the limb bud resulted in loss of *Shh* expression and reduced expression of SHH signaling targets, *Gli1* and *Ptch1*. Limb-specific overexpression of *Etv2* resulted in an ectopic expression of *Shh* and ectopic SHH signaling. This led to the development of preaxial polydactyly resembling human and animal mutations^22^. Furthermore, we demonstrated that known mutations of the ZRS associated with preaxial polydactyly resulted in enhanced binding of ETV2 to the ZRS. Based on these data, we propose that the collective dosage of ETV2 activity regulates the strength of ZRS-*Shh* system and contributes to the patterning and outgrowth of the limb bud.

Although previous analysis of ZRS activities using the *ZRS-LacZ* reporter transgenic mice at the *Shh* expression maintenance phase suggested that the ETS/ETV family TFs regulated the ZRS^27^, this report fell short of identifying the ETS/ETV TF that regulates ZRS. It was hypothesized that the collective activities of ETS/ETV factors, namely ETS1, ETS2, GABPa, ETV4 and ETV5 regulate *Shh* expression in the limb bud^27^. However, both *Ets1* and *Ets2* were dispensable for the development of the embryo proper^58–60^. Even when both *Ets1* and *Ets2* were deleted, embryos only exhibited endothelial cell defects and limbs developed normally^61^. *Gabpa* knockout animals also did not show limb defects (International Mouse Phenotyping Consortium, http://www.mousephenotype.org/data/genes/MGI:95610). Thus, previous studies did not identify the ETS/ETV family member responsible for *Shh* expression. ETV2 distinguishes itself from previously studied ETS/ETV family members in that loss or overexpression of *Etv2* alone affects limb development. Indeed, it is one of the few TFs along with *Hand2* that a single gene knockout causes severe limb defects. Furthermore, our transcriptional assays indicated that ETV2, but not ETS1 or ETS2, could activate transcription through ZRS. In this light, it is interesting to note a recent report that a mutation of ETV2 was linked to human polydactyly^62^. Although the effect of the mutation in the reported patient on ETV2 activity is not known, it underscores the key regulatory role of ETV2 in limb development.

Our analyses also revealed the antagonism of ETV2 and ETV4/5 in the transcriptional assay. It has been reported that loss of ETV function results in anterior expansion of *Shh*, suggesting that ETV4/5 is antagonizing the transcriptional activator of *Shh* during limb patterning^30^. Our data are consistent with the notion that ETV2 is the positive regulator of *Shh* that antagonizes ETV4/5 activity and establish the anterior-posterior identity of the digits.

Another distinguishing feature of ETV2 is that it is expressed earlier than *Shh* and that it regulates initiation phase of *Shh* expression. *Gabpa*, *Ets1*, and *Ets2* were expressed later than *Shh* in a broader population than the *Shh* expressing population in the limb bud and did not explain the establishment of the initial localized expression of *Shh*^27,63^. ETV2 fills this role. It is possible that ETV1, ETV2, and GABPa regulate expansion and maintenance of *Shh* expression through enhancers made accessible by ETV2.

Our third discovery was the definition of global chromatin accessibility changes according to limb development. Our ATAC-seq data and PCA of multiple samples revealed that there was a dramatic global change of chromatin accessibility between E9.5, E10.25, and E10.5, and between the ETV2-expressing and the non-expressing cells. Associated with the chromatin accessibility change, the profile of accessible TF binding sites changed globally. The known limb enhancers overlapped with the putative ETV2 binding sites that were closed in E9.5 HL and open in E10.5 posterior tissues, suggesting that the relaxation of chromatin accessibility at limb enhancers was correlated with relaxation of ETV2 binding sites not only at ZRS but also globally.

Genetic studies demonstrated that limb progenitors set up an anterior-posterior polarity during the initial 12 h from the onset of outgrowth^64^. This pre-patterning phase was necessary for the subsequent establishment of signaling centers, such as *Shh*-expressing ZPA and *Fgf8*-expressing AER^23,64^. ETV2-dependent global changes of chromatin accessibility coincide with this time window. This temporal correlation and the *Etv2*CKO phenotypes suggest that global changes of chromatin landscape triggered by ETV2 promoted the changes of transcriptional regulatory network from a pre-patterning mode to an organizing center-dependent patterning mode by priming the proliferative limb progenitors to be responsive to morphogen signals. Development of tissues and organs starts with specification of a small progenitor population, which subsequently undergoes patterning and proliferation for morphogenesis. Such global changes of chromatin landscape, which we demonstrated in limb progenitors, may be a common regulatory mechanism for progression of developmental processes in various organs.

**Fig. 7:**
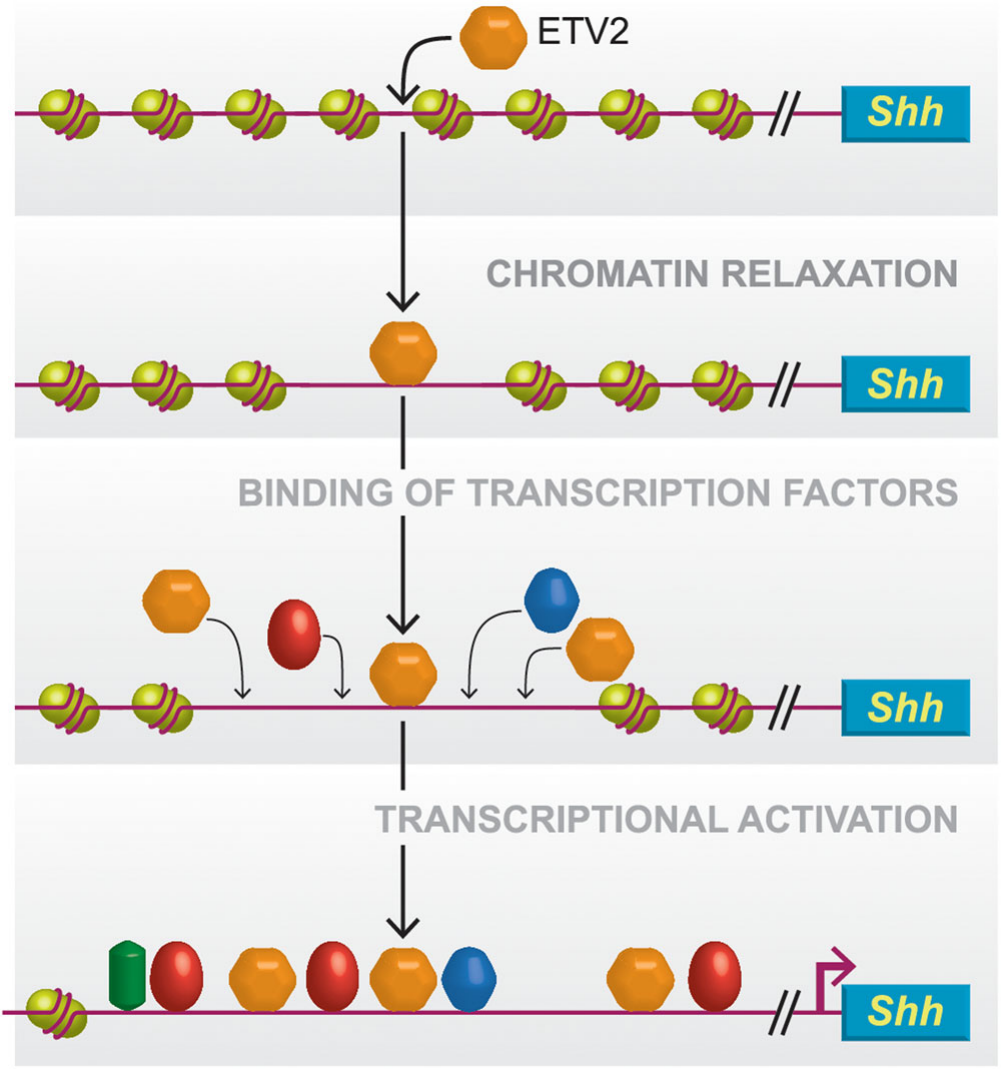
Proposed model of ETV2 function in activation of *Shh* expression. ETV2 functions in multiple steps of gene activation related to limb development. ETV2 depicted as an orange hexagon: (i) targets nucleosome-occupied regions and mediates nucleosome displacement; (ii) recruits additional transcription factors to the limb enhancers, including the ZRS, and facilitates transcription. ETV2 also (iii) activates transcription as a transcriptional activator. The dosage of ETV2 at the ZRS affects the expression levels and the domain of *Shh* expression and leads to polydactyly.

In summary, our studies demonstrated the essential role for ETV2 as a global epigenetic regulator and TF in the developing limb with *Shh* being a prominent target. We propose a model whereby ETV2 functions at multiple steps of gene activation (Fig. 7). ETV2 initially binds to nucleosome-occupied DNA, displaces nucleosomes, and relaxes the chromatin. This allows downstream factors to bind to the respective target sequences. ETV2 also binds to the target sequence and participates in transcriptional activation at the open chromatin region. We predict that such multi-step gene activation is a universal mechanism in regulation of tissue specification and patterning.

## Methods

### Animal assurance

The scientific merit, the ethical justification of the experimental studies and the animal welfare as outlined in the studies were reviewed and approved by the Institutional Biosafety Committee (Code number: 1010H90634) and the Institutional Animal Care and Use Committee (Protocol numbers: 1503-32435A, 1601-33438A, 2005-38150A) at the University of Minnesota.

### Mice

Animals were housed in a mouse facility with 12 h dark/light cycle, ambient temperature and humidity. Transgenic technologies were performed at the Mouse Genetics Laboratory at the University of Minnesota. The *Etv2* knockout mouse^37^, *Etv2-EYFP* reporter line^40^, *Etv2-Cre* transgenic line^40^, floxed *Etv2* allele (*Etv2*^fl/fl^)^65^ were previously described. The *Etv2-CreERT2* construct was generated by fusing the *CreERT2* coding region to the *Etv2* 3.9 kb regulatory region^39^ and generating a transgenic animal. *ROSA26-LacZ, ROSA26-ZsGreen1,* *ROSA26-STOP-rtTA-IRES-EGFP*, *Shh-EGFP-Cre*^16^, and *HoxB6-Cre*^41^ lines were obtained from the Jackson Laboratories. The *iHA-Etv2* transgenic line was generated by blastocyst chimera formation using ES cells that harbored a doxycycline-inducible *Etv2* expression cassette^66^.

For embryonic analysis, mice were bred, and timed pregnancies were generated. A posterior lateral plate- and limb mesoderm-specific knockout of *Etv2* was generated by crossing *HoxB6-Cre*^*Tg/Tg*^*; Etv2*^*+/−*^ males with *Etv2*^*fl/fl*^ females^65^. Conditional mutant mice (*HoxB6-Cre*^*Tg/+*^*;Etv2*^*fl/−*^, denoted as *Etv2*CKO) and heterozygous littermates (*HoxB6-Cre*^*Tg/+*^*;Etv2*^*fl/+*^, denoted as heterozygote) were compared.

For targeted overexpression of *Etv2* in limb mesoderm, first, a transgenic mouse harboring HA-tagged *Etv2* under the control of tetracycline-inducible promoter was generated from *iHA-Etv2* ES cells. This mouse was bred with the *HoxB6-Cre* driver line, and a compound heterozygous male (*HoxB6-Cre*^*Tg/+*^*; iHA-Etv2*^*Tg/+*^) was bred with *ROSA26-STOP-rtTA-IRES-EGFP* females to obtain triple transgenic embryos (*HoxB6-Cre*^*Tg/+*^*; iHA-Etv2*^*Tg/+*^*; ROSA26-STOP-rtTA-IRES-EGFP*^*Tg/+*^). At E9.5 and E10.5, pregnant dams were injected intraperitoneally with 25 mg/kg of doxycycline and harvested at E11.5 or 15.5 for analysis. In the triple transgenic embryos, rtTA and EGFP were expressed only in the *HoxB6-Cre* lineage, and the timing of HA-ETV2 expression could be controlled by administration of doxycycline (Dox). Littermates that do not carry the *iHA-Etv2* or the *rtTA-IRES-EGFP* transgenes were used as controls.

### Cell line

NIH3T3 cells were purchased from American Type Culture Collection (ATCC) (catalog number CRL-1658) and were cultured in Dulbecco’s Modified Eagle’s Medium with 10% calf serum with additives according to the protocol published by ATCC.

### Collection of limb mesenchymal cells and FACS sorting

Timed pregnant CD1 females bred with *Etv2-EYFP* males were sacrificed and embryos were collected in ice-cold PBS. Embryos were examined using epifluorescence microscopy and EYFP positive and negative embryos were separated. Limb buds were excised at the base of the limb buds and either pooled or processed separately. Limb bud tissues were digested in 200 μl of TripLE (ThermoFisher 12605010) at 37 °C in two cycles of 5 min incubation followed by trituration. Cells were cooled on ice for 5 min between incubations. Digestion was stopped with 1 ml of medium with 10% fetal calf serum that was filtered through a 50 μm mesh filter (Partec). Cells were collected by centrifugation and stained with staining medium containing 0.2 μg each of CD31-APC, Tie2-APC, CD45-APC and 0.5 μg anti CD16/CD32, and EYFP positive, APC negative live cells were sorted on FACSAria (BD). Cells from EYFP negative embryos were used as a negative control for FACS sorting.

### Chromatin Immunoprecipitation (ChIP) assay

ChIP was carried out according to the protocol of Mazzoni et al. (Mazzoni, Nature Methods vol8, No 12 2011) using the buffer system provided in the ChIP assay kit (Millipore). 1 × 10^6^ of dissociated cells were suspended in 200 μl of lysis buffer and processed according to the manufacturer’s protocol. Chromatin was sonicated with five 10 sec pulses at 20% power using Sonic Dismembrator model 500 (Fisher). Two μg of antibodies or control IgG was used for immunoprecipitation, and the chromatin-antibody complexes were separated using Dynabeads Protein G (Invitrogen). Crosslinking was reversed and recovered DNA was analyzed by qPCR corresponding to regions a, b, c, and d of ZRS (Fig. 4A) and the promoter region of the GAPDH gene. After the qPCR reaction, the PCR products were analyzed on a gel to confirm the proper amplification of the targeted region and presence or absence of the PCR products. See Supplementary Table 3 for oligonucleotide sequences.

### Immunohistochemistry

Immunohistochemical analyses of sections were performed as previously described^40^. All limb sections were made transversely (in the direction perpendicular to the rostro-caudal body axis) unless noted otherwise. Antibodies and dilutions used are listed in Supplementary Table 1. Images were obtained using a Nikon C2 Upright Confocal Microscope at the University Imaging Center or Zeiss LSM710 Confocal Microscope at the Developmental Biology Center at the University of Minnesota-Twin Cities. Z-stack images were obtained in 10 μm steps in separate channels and digitally merged.

### Skeletal preparation and in situ hybridization

Alcian Blue skeletal staining and whole mount in situ hybridization were performed as previously described^67^.

### TUNEL staining

Sections were prepared and stained using in situ cell death detection kit (Roche) for TUNEL staining.

### qRT-PCR and In situ hybridization analyses

RNA extraction was performed using the Qiagen Micro RNeasy kit (Qiagen) according to the manufacturer’s protocols. qRT-PCR was carried out using SuperScript III Reverse Transcriptase (ThermoFisher) and Taqman probes (ThermoFisher). Three biological replicates were prepared for each experiment and each sample was analyzed by real-time PCR in triplicate. Whole mount in situ hybridization was performed as previously described^68^.

### ATAC-PCR, ATAC-seq data processing, and chromVAR analysis

Cells were prepared as described in the FACS procedure. ATAC reaction was performed as previously described using the Tn5 transposase (Illumina) and libraries were created at the University of Minnesota Genome Center^69^. For ATAC-PCR analysis, 0.94 ng of the library was amplified in each well with primer pairs used for ChIP analysis (Fig. 4A). The GAPDH promoter region was used as an internal control, and all amplification rate is shown relative to the amplification of the GAPDH promoter. All libraries were sequenced using 75-bp paired end sequencing on MiSeq (Illuminia). The cells with less than 100 K paired reads were removed. The raw reads were mapped to mouse genome (mm10) using Bowtie2 (v2.2.4), with the parameters -X2000 and -m1, to ensure that the fragments up to 2 kb were allowed and that only unique aligning reads were retrieved^70,71^. The duplicates were removed by Picard. To adjust the read start sites to represent the center of the transposon binding, all reads aligning to the plus strand were offset by  + 4 bp, and all reads aligning to the -strand were offset by −5 bp. For each ATAC-seq sample, we first summarized the coverage at each genomic locus, followed by slicing the genome into discrete peak regions where the minimum reads coverage at each locus is at least 10. These peaks were extended to 500 bp intervals centered at the summit. The 500 bp peak intervals from individual ATAC-seq sample were combined into a union set of 536,981 intervals and used for the downstream chromVAR analysis. The chromVAR analysis was performed by using 332 TF motifs from Homer database (https://github.com/GreenleafLab/chromVARmotifs). The deviation scores and the TF variability were derived by using chromVAR’s computeDeviations() and computeVariability() functions. The pathway analysis was performed using peaks clustered together with the ZRS region genomic interval, and were annotated using ChIPseeker^72^. The geneID obtained from ChIPseeker was used as the gene set to perform GO enrichment analysis using clusterProfiler^73^ to obtain enriched pathways.

### SeATAC analysis

We used a variational autoencoder model implemented in R package SeATAC (v0.4.0, https://github.com/gongx030/seatac)^50^ for the detection of genomic regions with differential chromatin accessibility and nucleosome positions. SeATAC employed a conditional variational autoencoder framework to model the ATAC-seq-specific V-plot while addressing the batch effect in the ATAC-seq data, allowing an unbiased comparison across multiple samples. The convolutional neural network (CNN) blocks used in the encoder network allowed SeATAC to robustly estimate the posterior distribution of the latent variables by considering ATAC-seq specific fragment size profile, resulting in superior performance on several tasks such as detecting differential V-plot, recovering nucleosome positions, detecting nucleosome changes and calling TFBS with differential chromatin accessibility.

We modeled the V-plot ${{{{{{\boldsymbol{x}}}}}}}_{{ni}}$ of each genomic region $i$ in each sample $n$ as a probabilistic distribution $p\left({{{{{{\boldsymbol{x}}}}}}}_{{ni}}|{{{{{{\boldsymbol{z}}}}}}}_{{ni}},{s}_{n}\right)$ conditioned on the sample indicator ${s}_{n}$ of each sample, as well as an unobserved latent variable ${{{{{{\boldsymbol{z}}}}}}}_{{ni}}$. The sample indicator ${s}_{n}$ represents the nuisance variation due to the sample-specific fragment size profile. The latent variable ${{{{{{\boldsymbol{z}}}}}}}_{{ni}}$ is a $K$ dimensional vector of Gaussians representing the remaining variation with respect to the underlying V-plot ($K=5$). In SeATAC, a neural network serves as a decoder to map the latent variables ${z}_{{ni}}$ and sample indicator ${s}_{n}$ to an estimated output V-plot. We expected that latent variables provide batch-corrected representations of the V-plot for the differential analysis. We derived an approximation of the posterior distribution of the latent variable $q\left({{{{{{\boldsymbol{z}}}}}}}_{{ni}}|{{{{{{\boldsymbol{x}}}}}}}_{{ni}}{{{{{\boldsymbol{,}}}}}}{s}_{n}\right)$ by training another encoder neural network using variational inference and a scalable stochastic optimization procedure. The variational distribution $q\left({{{{{{\boldsymbol{z}}}}}}}_{{ni}}|{{{{{{\boldsymbol{x}}}}}}}_{{ni}}{{{{{\boldsymbol{,}}}}}}{s}_{n}\right)$ is chosen to be Gaussian with a diagonal covariance matrix, where the mean and covariance are estimated by an encoder neural network applied to $\left({{{{{{\boldsymbol{x}}}}}}}_{{ni}}{{,}}{s}_{n}\right)$. The variational evidence lower bound (ELBO) is

$${{{{{\rm{log }}}}}}\,p\left(x|s\right)\ge {{\mathbb{E}}}_{q\left(z|x,s\right)}{{{{{\rm{log }}}}}}\,p\left(x|z,s\right)-{D}_{{KL}}\left[q\left(z|x,s\right)\parallel p(z)\right].$$

A standard multivariable normal prior $p({{{{{{\boldsymbol{z}}}}}}}_{{ni}})$ is used in SeATAC because it can be reparametrized in a way that allows backpropagation to flow through the deterministic nodes. To optimize this lower bound we used the reparameterization trick to compute low-variance Monte Carlo estimates of the expectations’ gradients. Throughout the study, we used Adam optimizer (learning rate = 0.01) with a cosine learning rate schedular with warmup.

### Electrophoretic mobility shift assay

pcDNA3.1-Etv2-HA or empty pcDNA3.1(+)-HA vectors were expressed using the TNT Quick Coupled Transcription/Translation System (Promega, Madison, WI) according to the manufacturer’s protocol. DNA oligonucleotides corresponding to wild-type ZRS sequence or ZRS sequence with combinations of inactivating mutations in putative ETS, HAND2, or HOXD13 binding sites (see Supplementary Table 3 for oligonucleotide sequences) were synthesized with and without the IRDye® 700 fluorophore (Integrated DNA Technologies, Coralville, IA). Complimentary WT or mutant oligonucleotides were annealed to generate labeled probe and unlabeled competitor DNA. In vitro synthesized protein (1 µL) was incubated with 250 ng of poly dI-dC in binding buffer (50 mM Tris pH 7.6, 80 mM NaCl, 8% glycerol) at room temperature for 10 min. For antibody treatment, pre-binding was performed in the presence of 0.2 µg of active or heat-inactivated anti-human ETV2 antibody, anti Hand2 antibody, or anti HoxD13 antibody (see Supplementary Table 1 for antibody information). IRDye® 700-labelled probe (100 fmol) was then added and the binding reaction proceeded at room temperature for 15 min. DNA-protein complexes were resolved on a 6% non-denaturing polyacrylamide gel in 0.5x TBE (40 mM Tris pH 8.3, 45 mM boric acid, and 1 mM EDTA) at room temperature. Fluorescence was detected using an Odyssey CLx imager (LI-COR Biosciences, Lincoln, NE).

### Luciferase reporter assay

NIH3T3 cells were plated at 20000 per well of a 48 cluster plate overnight and transfected on the next day using Lipofectamine 3000 Reagent (ThermoFisher). After 24 h of incubation, cells were washed in PBS and Luciferase activity was measured using Dual-Luciferase ® Reporter Assay System (Promega). Each sample was transfected in quardruplicate, and experiments were repeated at least three times to confirm reproducibility. Empty vector was added to equalize the total amount of DNA transfected when applicable.

### Reporter plasmids

Genomic DNA fragments corresponding to regions i (1-656), ii (657-1401), and iii (1402-1682) of ZRS were amplified from pGL4-TATA-ZRS^23^ by PCR (see Supplementary Table 3 for primer sequences) and subcloned into the *Kpn*I-*Bam*HI fragment including the vector backbone of pGL4-TATA-ZRS^23^.

### Quantification and statistical analysis

Data represent average of more than three replicates (replicate numbers are indicated in the figure legends) and SEM. Statistical tests used are indicated in the figure legends. *P*-values lower than 0.01 were determined to be significant. The above analyses were done with Prism 4.0 software (GraphPad Software).

For quantitative PCR analysis, at least three biological replicates were prepared for each experiment, and samples were analyzed using real-time PCR in triplicate. Transcription assays were performed in triplicate, and each experiment was repeated at least three times.

### Reporting summary

Further information on research design is available in the Nature Research Reporting Summary linked to this article.

## Supplementary information

Supplementary Information

Reporting Summary

## Data Availability

The code and datasets for the analysis can be found at https://github.com/gongx030/Etv2_limb_manuscript. The ATAC-seq data have been deposited at the Gene Expression Omnibus under the accession number GSE192865. Source data are provided with this paper. Further information and requests for resources and reagents should be directed to and will be fulfilled by the Lead Contact, Daniel J. Garry.

## Source data

Source Data
