# Supplementary material for: Etv2 regulates enhancer chromatin status to initiate Shh expression in the limb bud
Source: Nat Commun. 2022 Jul 21;13:4221. doi: 10.1038/s41467-022-31848-6 (PMC9304341; doi:10.1038/s41467-022-31848-6)
Supplement: Supplementary file 1 — Supplementary Information [file 41467_2022_31848_MOESM1_ESM.pdf]

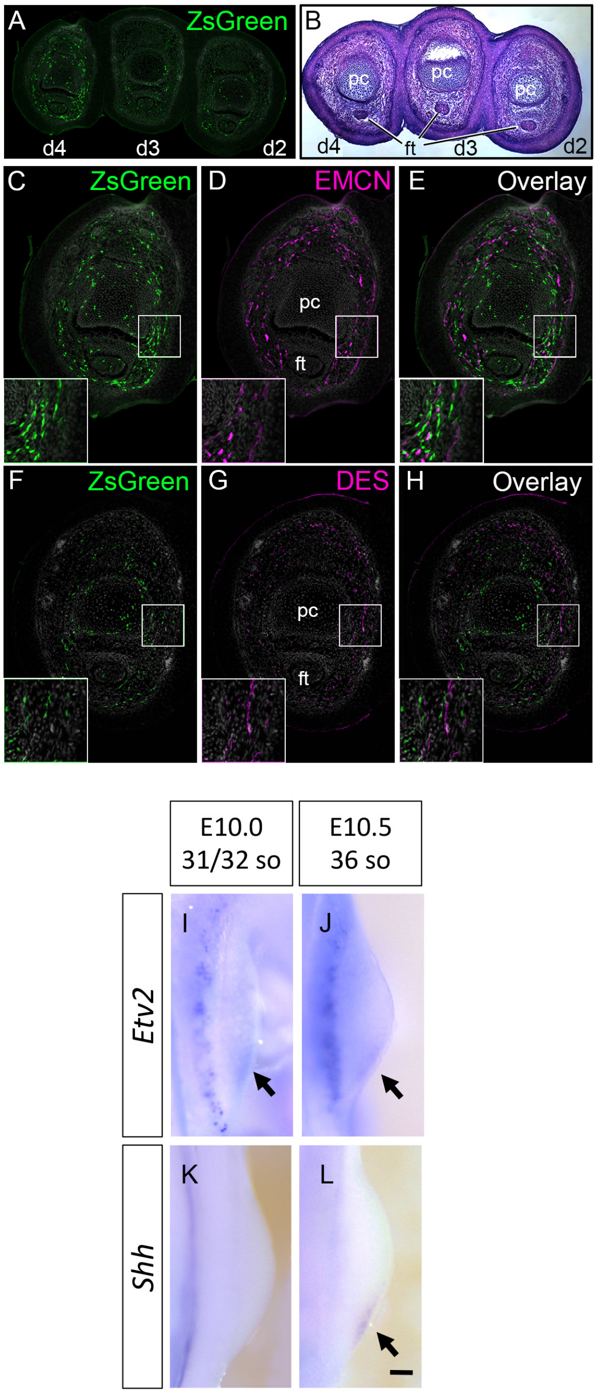
A
ZsGreen
d4
d3
d2
B
pc
pc
pc
d4
ft
d3
d2
C
ZsGreen
D
EMCN
pc
ft
E
Overlay
F
ZsGreen
G
DES
pc
ft
H
Overlay
E10.0
31/32 so
E10.5
36 so
Etv2
I
J
Shh
K
L

Figure S1 (related to Figure 1). The *Etv2* lineage contributes mainly to two posterior digits and does not co-label with endomucin or desmin. The E18.5 limb shown in Figure 1B and 1C was sectioned and analyzed. **(A, C-H)** Immunohistochemistry with GFP antibody, which detects ZsGreen (A, C, F), endomucin antibody (EMCN) (D), and desmin antibody (DES) (G). E and H are overlays of C&D and F&G, respectively. Boxed areas in each panel are enlarged in the bottom left corners. DAPI stain is shown in gray. (B) Hematoxylin-Eosin stain. d2-d4, digits 2-4; pc, primordial cartilage; ft flexor tendon. **(I-L)** *In situ* hybridization of *Etv2* (I, J) and *Shh* (K, L) at the indicated embryonic stages. Somite numbers of each embryo are also indicated. Arrows in I, J and arrow in L indicate *Etv2* and *Shh* expression in hindlimb buds, respectively. At least three embryos were analyzed at each stage for each marker with essentially the same results.

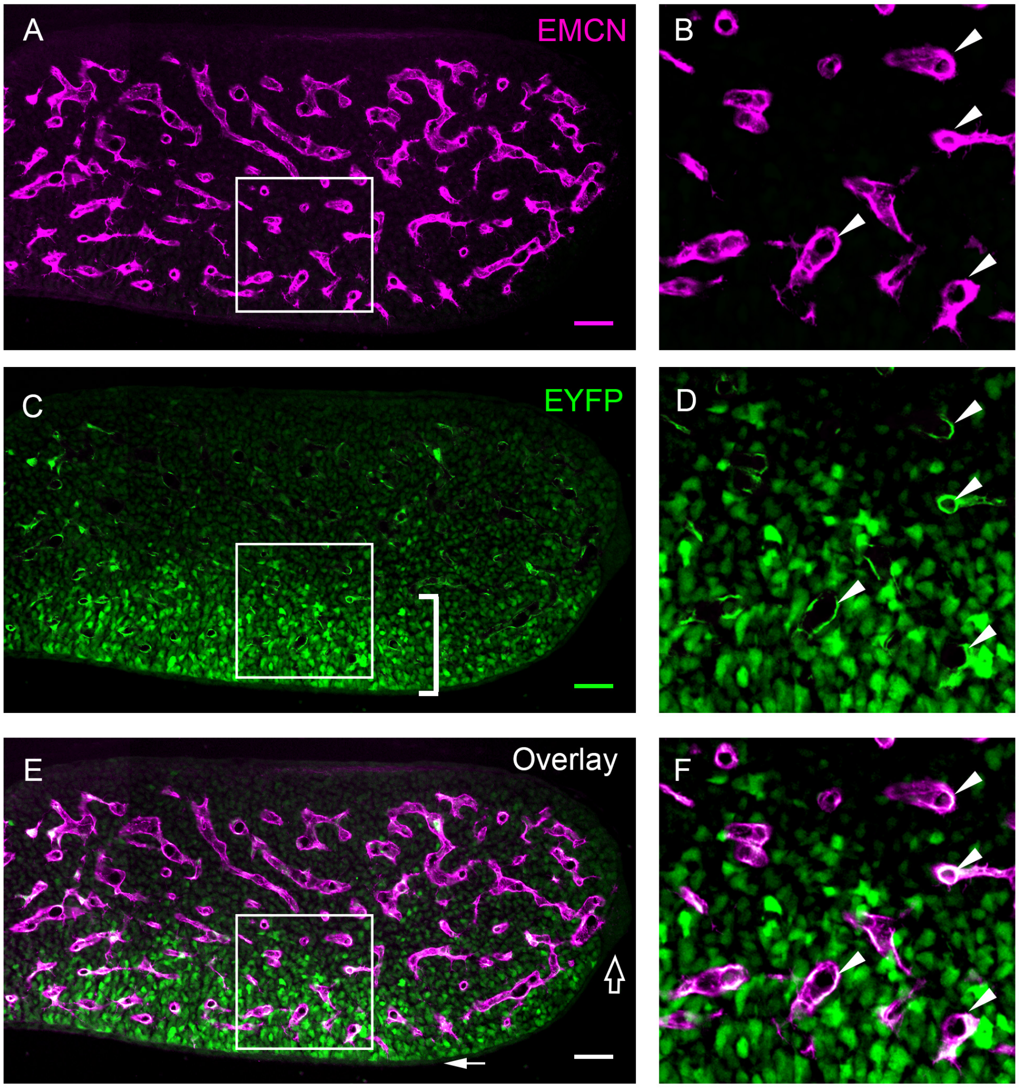
A
EMCN
B
C
EYFP
D
E
Overlay
F

Figure S2 (related to Figure1). ETV2 is expressed in two distinct populations of the developing limb. E10.5 forelimb from an *Etv2-EYFP* transgenic embryo was sectioned transversely (perpendicular to the rostro-caudal body axis) and analyzed by immunohistochemistry for ENDOMUCIN (EMCN) (A, B) and EYFP (C, D) expression. E and F are overlays of ENDOMUCIN and EYFP staining. Boxed areas in A, C, and E are enlarged in B, D, and F. Note that EYFP was expressed in two distinct populations: the endothelial lineage that expresses ENDOMUCIN (B, D, F, white arrowheads); and a non-endothelial population that was localized to ventro-posterior region of the limb bud (bracket in C). Note that EYFP was not expressed in the ectoderm (white arrow in E), including the apical ectodermal ridge (open arrow in E). Each analysis was repeated with three embryos with essentially the same results. Scale bar is 100 μm.

-

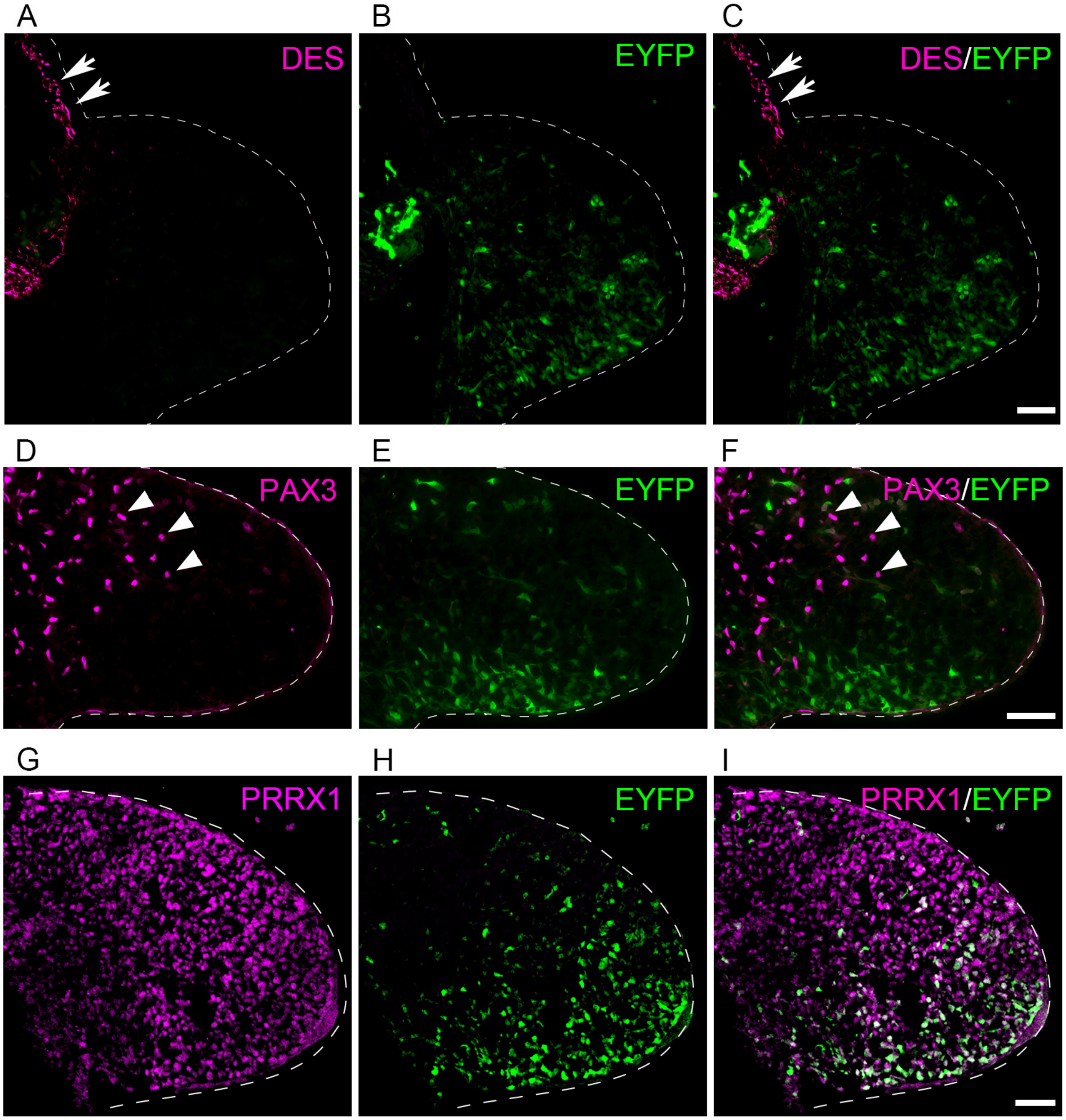
A
DES
B
EYFP
C
DES/EYFP
D
PAX3
E
EYFP
F
PAX3/EYFP
G
PRRX1
H
EYFP
I
PRRX1/EYFP

Figure S3 (related to Figure 1). ETV2 is expressed in the limb mesenchyme. (A-I) Cross sections of the E10.5 hindlimb buds of *Etv2-EYFP* transgenic embryos were made and analyzed by immunohistochemistry. Antibodies directed to DESMIN (A, C), PAX3 (D, F), PRRX1 (G, I), and GFP, which detects EYFP (B, C, E, F, H, I) were used. Broken lines indicate the outlines of the tissues. Note that EYFP did not colocalize with DESMIN (A, C, arrows) or PAX3 (D, F, arrowheads), but colocalized with PRRX1 (G, I). Scale bars are 100 μm. Each analysis was repeated with three embryos with essentially the same results.

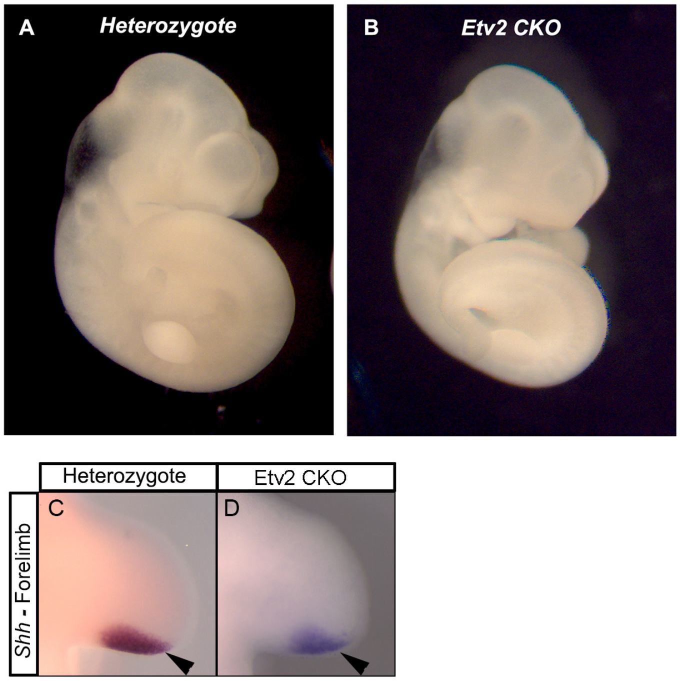

Figure S4 (related to Figure 2). Morphology of the control and *HoxB6-Etv2*CKO embryos. **(A, B)** Whole-mount images of control (A) and *Etv2*CKO embryos (B) at E10.5 are shown. **(C, D)** Whole-mount in situ hybridization of *Shh* at E10.5 (36-37 somites). Forelimb buds of the embryos in Figure 2A (control) and 2B (*Etv2*CKO) are shown. Note, in contrast to hindlimb buds that completely lacked *Shh* expression (Fig. 2B), *Shh* was present in forelimb buds. Five embryos were analyzed with essentially the same results.

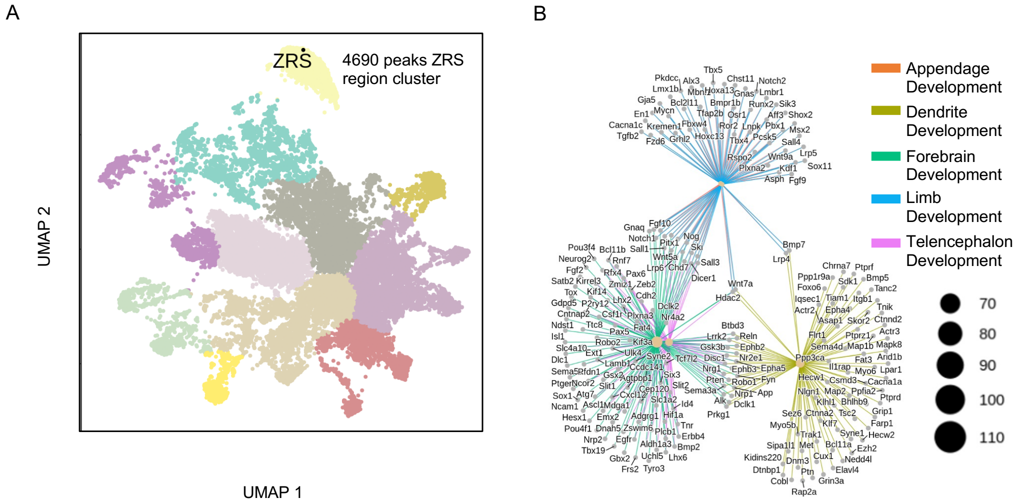

Figure S5 (related to Figure 3). ZRS belonged to a cluster with 4,690 ATAC-seq peaks, which nearby genes were significantly associated with limb development. (A) UMAP of among 110,133 200-bp ATAC-seq peaks across all samples. (B) The pathway analysis of the cluster of 4,690 ATAC-seq peaks, including ZRS.

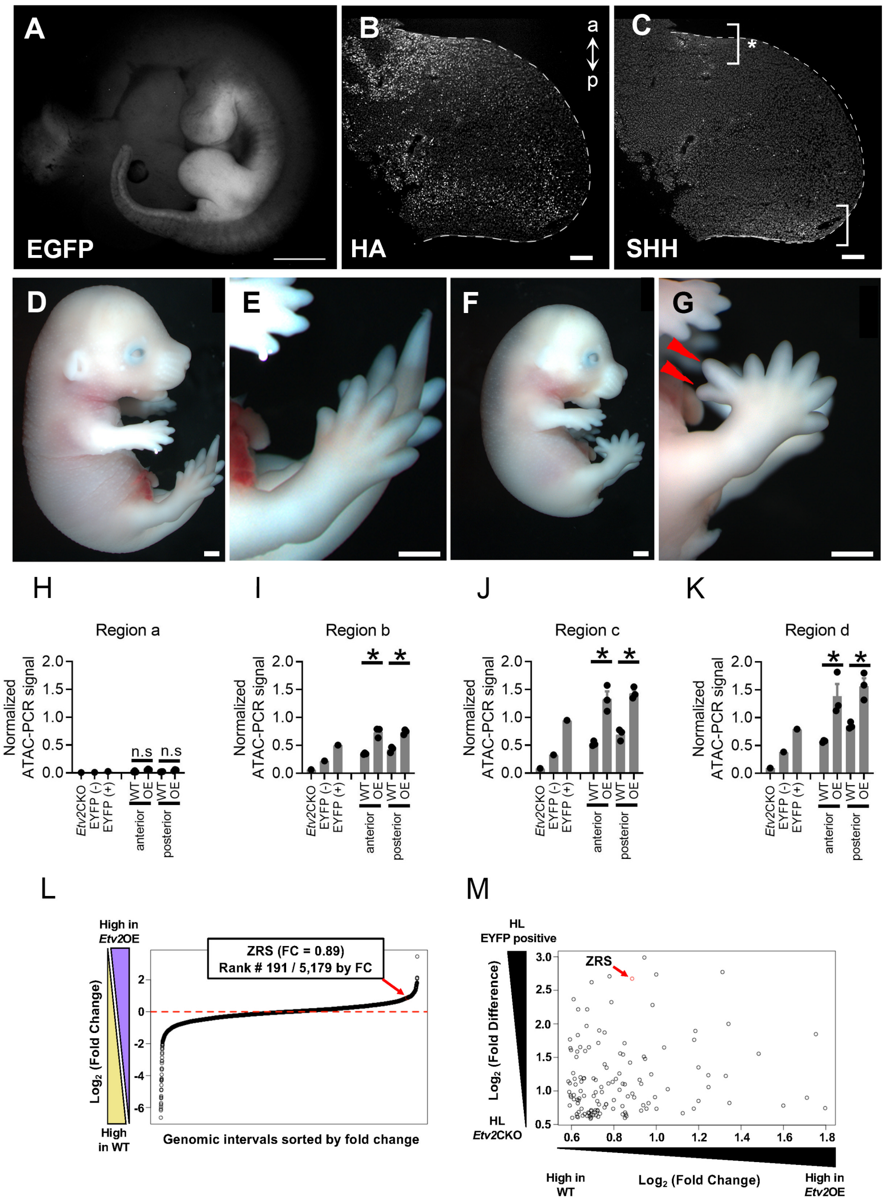
A
EGFP
B
HA
a
p
C
*
SHH
D
E
F
G
H
Region a
Normalized ATAC-PCR signal
n.s
n.s
Etv2CKO
EYFP (-)
EYFP (+)
WT
OE
WT
OE
anterior
posterior
I
Region b
J
Region c
K
Region d
L
High in Etv2OE
ZRS (FC = 0.89)
Rank # 191 / 5,179 by FC
Log2 (Fold Change)
High in WT
Genomic intervals sorted by fold change
M
HL EYFP positive
ZRS
Log2 (Fold Difference)
HL Etv2CKO
High in WT
Log2 (Fold Change)
High in Etv2OE

Figure S6 (related to Figure 5). Overexpression of *Etv2* in the hindlimb bud alters chromatin accessibility and results in polydactyly. **(A-C)** *HoxB6-Cre$^{Tg/+}$; iHA-Etv2$^{Tg/+}$; ROSA26-rtTA-IRES-EGFP$^{Tg/+}$* embryo was pulsed with Dox at E9.5 and E10.5 and analyzed at E11.5. Whole-mount analysis of EGFP epifluorescence at E11.5 confirms Cre recombination in the hindlimb (A). A frontal section (sectioned perpendicular to the dorso-ventral axis) of the hindlimb stained with antibody to HA epitope (B) and SHH protein (C). Broken lines indicate the outline of the section. Bars indicate 1mm in A and 100μm in B and C, respectively. The anterior (a)-posterior (p) direction is indicated by an arrow in B. Note the ectopic expression of SHH in the anterior domain (bracket with asterisk in C), in addition to the normal expression domain (bracket in C). **(D-G)** Whole-mount images of embryos shown in Figure 5D and 5H. **(H-K)** ATAC-PCR analysis of the limb bud samples used for ATAC-seq. Chromatin accessibility of regions a (H), b (I), c (J), and d (K) were assessed by qPCR relative to the accessibility of the GAPDH promoter. Samples analyzed are: from left to right, *Etv2*CKO hindlimb, hindlimb *Etv2*-EYFP negative, hindlimb *Etv2*-EYFP positive (samples used in Figure 3D), wild type anterior half of the hindlimb, *Etv2*OE anterior half of the hindlimb, wild type posterior half of the hindlimb, and *Etv2*OE posterior half of the hindlimb (samples used in Figure 5I). *Etv2*CKO hindlimb, hindlimb *Etv2*-EYFP negative, hindlimb *Etv2*-EYFP positive bars are qPCR of an ATAC-seq library generated with pooled limb buds from 5, 20, and 20 embryos, respectively. Wild type and *Etv2*OE bars each represent an average of the qPCR data obtained from three ATAC-seq libraries generated from three independent embryos. Data in the graphs are presented as mean values +/- SEM. Statistical test: one-way ANOVA with Holm-Sidak's multiple comparisons test. Note that the chromatin of Region a is closed in all samples (H), but Regions b-d are more accessible, and the accessibility was increased by overexpression of *Etv2* (I-K). (L) Genomic intervals ranked by the degree of accessibility change according to overexpression of *Etv2*. (M) Plot of accessibility difference between (hindlimb EYFP positive/*Etv2*CKO) versus (*Etv2*OE anterior/ wildtype anterior).

Supplementary Table 1. Antibodies

ANTIBODIES	SOURCE	IDENTIFIER
Anti Active Caspase-3 clone C92-605 (1:200 for immunohistochemistry)	BD Biosciences	BD Cat#559565, RRID:AB_397274
Anti Desmin (1:250 for immunohistochemistry)	Novus	Novus Cat# NB120-15200, RRID:AB_789575
Anti E47 (0.2μg/reaction for EMSA)	Santa Cruz	Cat# sc-763X, RRID:AB_631405
Anti Endomucin (1:400 for immunohistochemistry)	Abcam	Cat# ab106100, RRID:AB_10859306
Anti Etv2 (0.2μg/reaction for EMSA, 2μg/reaction for ChIP)	Sana Cruz	Cat# sc-164278, RRID:AB_2100842
Anti GFP (1:400 for immunohistochemistry)	Abcam	Cat# ab13970, RRID:AB_300798
Anti HA (1:200 for immunohistochemistry)	Roche	Cat# 11867423001, RRID:AB_390918
Anti Hand2 (0.2μg/reaction for EMSA)	Santa Cruz	Cat# sc-9411, RRID:AB_2115993
Anti HoxD13 (0.2μg/reaction for EMSA)	Santa Cruz	sc-46364X
Anti Pax3 (1:200 for immunohistochemistry)	DSHB	Cat# PAX3, RRID:AB_528426
Anti phospho histone H3 (1:200 for immunohistochemistry)	Millipore	Cat# 05-806, RRID:AB_310016
Anti Prrx1 (1:100 for immunohistochemistry)	Abcam	Ab211292
Anti Smooth Muscle Actin (1:800 for immunohistochemistry)	Thermo	MS-1B-90
Anti-SHH (1:200 for immunohistochemistry)	Santa Cruz	Cat# sc-9024, RRID:AB_2239216
Control mouse IgG (0.2μg/reaction for EMSA)	Jackson	Cat# 015-000-003, RRID:AB_2337188
Control goat IgG (0.2μg/reaction for EMSA, 2μg/reaction for ChIP)	Jackson	Cat# 005-000-003, RRID:AB_2336985
Control Rabbit IgG (0.2μg/reaction for EMSA)	Jackson	Cat# 011-000-003, RRID:AB_2337118
Tie2-PE (0.2μg per million cells for FACS)	e-bioscience	Cat# 12-5987-83, RRID:AB_466101
CD31-PE (0.2μg per million cells for FACS)	Becton Dickinson	Cat# 553373, RRID:AB_394819
CD45-PE (0.2μg per million cells for FACS)	e-bioscience	Cat# 12-0451-82, RRID:AB_465668
CD16/CD32 (0.5μg per million cells for FACS)	Becton Dickinson	Cat# 553141, RRID:AB_394656

Supplementary Table 2. Key Resources

Chemicals, Peptides, and Recombinant Proteins		
4-hydroxy tamoxifen	Sigma-Aldrich	H7904; CAS:68047-06-3
doxycycline	Sigma-Aldrich	D3447; CAS:10592-13-9
Critical Commercial Assays		
Qiagen Micro RNeasy kit	Qiagen	74004
In situ cell death detection kit, TMR red	Roche	12156792910
Nextera Tn5 transposase	Illumina	FC-121-1030
Qiagen MinElutePCR purification kit	Qiagen	28004
SsoAdvanced SYBR Green Supermix	Bio-Rad	172-5260
ChIP assay kit	Millipore	17-295
Dynabeads Protein G	Invitrogen	10003D
Dynabeads Protein A	Invitrogen	10001D
Click-iT^{TM} Alexa Fluor^{TM}488 Imaging Kit	ThermoFisher	C10337
Deposited Data		
GEO accession numbers for ATAC-seq data	GSE192865	
Token for Reviewer access to the data	mxwtmiyelporjon	
Experimental Models: Cell Lines		
Mouse: NIH3T3 cells	ATCC	CRL-1658
Experimental Models: Organisms/Strains		
Etv2 knockout	Ferdous et al., 2009	N/A
Etv2$^{flox/flox}$	Shi et al., 2015	N/A
Etv2(ER71)-EYFP	Rasmussen et al., 2011	N/A
Etv2(ER71)-Cre	Rasmussen et al., 2011	N/A
Etv2(ER71)-CreERT2	This paper	N/A
ROSA26-LacZ	Freidrich 1 et al., 991	RRID:IMSR_JAX:003309
ROSA26-ZsGreen1	Madisen et al., 2010	RRID:IMSR_JAX:007906
ROSA26-rtTA-ires-EGFP	Belteki et al., 2005	RRID:IMSR_JAX:005670
iHA-Etv2	This paper/ Behrens et al. 2014	N/A
HoxB6-Cre	Lowe et al., 2000	RRID:IMSR_JAX:017981
Shh-EGFP-Cre	Harfe et al., 2004	RRID:IMSR_JAX:005622
Oligonucleotides		
See Supplementary Table 3 for oligonucleotides used for ChIP assay, EMSA, and DNA construction	Supplementary Table 2	N/A

Recombinant DNA		
ZRS reporter plasmid: pGL4-TATA-ZRS-Luciferase	Galli et al., 2010	N/A
Internal control plasmid for luciferase assay: pRL-CMV	Promega	E2231
Control plasmid for transfection: pCDNA3-HA	Ferdous et al., 2009	N/A
Flt1 reporter plasmid: pGL3-Flt1-Luciferase	Koyano-Nakagawa et al., 2015	N/A
HoxD13 plasmid: pCDNA3.1(+) 3-Flag hu HoxD13 (H4-1)	This paper	N/A
Hand2 plasmid: TnT (M60-3)	This paper	N/A
Ets1 plasmids: pCDNA3 C-term 1X flag Ets1 (M2-11) for protein synthesis, pCMV-SPORT6-Ets1 for transfection	This paper	N/A
Ets2 plasmids: pCDNA3 C-term 1X flag (M2-2) for protein assay, pCMV-SPORT6-Ets2 for transfection	This paper	N/A
Etv4 plasmid: pQCXiH-Etv4	Hollenhorst et al., 2011	N/A
Etv5 plasmid: flag tagged Etv5	Zhang et al., 2010	N/A
Etv2 plasmids: pCDNA HA-ER71XS for protein assay, pCSFmt107-ER71 for transfection	This paper/Koyano-Nakagawa et al, 2012	N/A
Shh plasmid for in situ hybridization	Akiyama et al., 2015	N/A
Ptch1 plasmid for in situ hybridization	Hayashi et al., 2016	N/A
Gli1 plasmid for in situ hybridization	Akiyama et al., 2015	N/A
Hand2 plasmid for in situ hybridization	Hayashi et al., 2016	N/A
Hoxd13 plasmid for in situ hybridization	Hayashi et al., 2016	N/A
Dusp6 plasmid for in situ hybridization	Kawakami et al., 2003	N/A
Nmyc plasmid for in situ hybridization	Akiyama et al., 2015	N/A
Prrx1 plasmid for in situ hybridization: pCR-II-Prx1 (M1-2)	This paper. Sequence below was cloned into pCR-II (Invitrogen)	N/A
GCCGGGGACA TGGTGGCGGC ACAAGCAGAC GAAAGTGTGG GCGAGGCGGG CCGGAGCCTG CTGGAGTCAC CGGGACTGAC CAGTGGCAGC GACACCCCTC AGCAGGACAA TGACCAGTTG AACTCTGAGG AGAAGAAGAA GAGAAAGCAG CGGAGAAACA GGACAACATT CAACAGCAGC CAACTGCAGG CCTTGGAGCG TGTCTTTGAG CGGACACATT ACCCGGATGC TTTTGTTCGA GAAGATCTCG CACGTCGGGT GAACCTCACT GAGGCCAGAG TGCAGGTGTG GTTTCAGAAC CGAAGAGCCA AGTTCCGCAG GAATGAGCGA GCCATGCTGG CCAATAAAAA CGCTTCTCTC CTCAAGTCCT ACTCAGGAGA CGTGACTGCT GTGGAGCAAC CCATCGTACC TCGTCCTGCT CCCAGACCAA CCGATTATCT CTCCTGGGGG ACAGCCTCTC CGTACAGCGC CATGGCTACT TATTCTGCCA CATGTGCCAA CAATAGCCCT GCACAGGGTA		
Software and Algorithms		
Statistical analysis	GraphPad	Prism 7
Other (qRT-PCR reagents)		
Absolute Blue qPCR Rox Mix	Thermo	AB-4138
Etv2	ABI	Mm00468389_m1

Shh	ABI	Mm00436528_m1
GAPDH	ABI	4351309

Supplementary Table 3. Oligonucleotides

Oligonucleotides	Sequence	ID
ChIP Region a, sense	TTCGTTTGATGACTAAATGAGGTAAT	678
ChIP Region a, antisense	TCTCCTTATAAATTGCAGGTCTAAAAA	679
ChIP Region b, sense	ACACATGATCTATAGGATTAAGAG	670
ChIP Region b, antisense	CTATTGTGCTGTCATGTTGCTTGG	671
ChIP Region c, sense	GTCACAGTTTGAGATTGTCCTGGT	674
ChIP Region c, antisense	TGAAAGAATCCAATGAACGCTCATG	675
ChIP Region d, sense	GGCAAATGCGCAAACTCAGTCTG	676
ChIP Region d, antisense	TCTTGCAGGTGTTGGGAGAATCA	677
ChIP GAPDH promoter, sense	CCCTTTTCTGCCTTCCTACC	658
ChIP GAPDH promoter, antisense	TGCTGAAGTGCTCCCTACCT	659

Oligonucleotides for EMSA	Sequence (Mutated nucleotides are indicated in lower case)	ID
Ets#1, upper strand	CTGAGGTCACTTCCTCTCTTAATT	814
Ets#1, lower strand	AATTAAGAGAGGAAGTGACCTCAG	815
Ets#1, upper strand, mutated	CTGAGGTCACTTgaaCTCTTAATT	818
Ets#1, lower strand, mutated	AATTAAGAGttcAAGTGACCTCAG	819
Ets#2, upper strand	GAAGAGAGTAGGAAGTCCAGCCTG	789
Ets#2, lower strand	CAGGCTGGACTTCCTACTCTCTTC	790
Ets#2, upper strand, mutated	GAAGAGAGTttcAAGTCCAGCCTG	634
Ets#2, lower strand, mutated	CAGGCTGGACTTgaaACTCTCTTC	635
Ets#3, upper strand	GAGCGATTCAGGAAGTGCTGCTTA	791
Ets#3, lower strand	TAAGCAGCACTTCCTGAATCGCTC	792
Ets#3, upper strand, mutated	GAGCGATTCttcAAGTGCTGCTTA	636
Ets#3, lower strand, mutated	TAAGCAGCACTTgaaGAATCGCTC	637
Ets#4, upper strand	CTGGGTGAAAGGAAATCACAGGCA	793
Ets#4, lower strand	TGCCTGTGATTTCCTTTCACCCAG	794
Ets#4, upper strand, mutated	CTGGGTGAAttcAAATCACAGGCA	638
Ets#4, lower strand, mutated	TGCCTGTGATTTgaaTTCACCCAG	639
Ets#5, upper strand	AACCTTGCAAGGAAATTTGACTTG	795
Ets#5, lower strand	CAAGTCAAATTTCCTTGCAAGGTT	796
Ets#5, upper strand, mutated	AACCTTGCAttcAAATTTGACTTG	640
Ets#5, lower strand, mutated	CAAGTCAAATTTgaaTGCAAGGTT	641
Ets#1-HOX-E, upper strand	ACTTTCATAATAAAAGTAAAATGCACAA AATCTGAGGTCACTTCCTCTCTTAATTA GTTGCACTGACCAGGTGGAGGCGAAG	650

Ets#1-HOX-E, lower strand	CTTCGCCTCCACCTGGTCAGTGCAACT AATTAAGAGAGGAAGTGACCTCAGATT TTGTGCATTTTACTTTTATTATGAAAGT	651
ETVA, upper strand	GTTTTAATATGTTTCTATCCTGTGT	626
ETVA, lower strand	ACACAGGATAGAAACATATTAAAAC	627
ETVB, upper strand	TATTACAGAAAATGAAGTCATATC	797
ETVB, lower strand	GATATGACTTCATTTTCTGTAATA	798
ETVA, Belg2 mutant, upper strand	GTTTTAATATGTTTCcATCCTGTGT	803
ETVA, Belg2 mutant, lower strand	ACACAGGATgGAAACATATTAAAAC	804
ETVB, AC mutant, upper strand	TATTACAGgAAATGAAGTCATATC	799
ETVB, AC mutant, lower strand	GATATGACTTCATTTcCTGTAATA	800
ETVB, Aus mutant, upper strand	TATTACAGAAAAgGAAGTCATATC	801
ETVB, Aus mutant, lower strand	GATATGACTTCcTTTTCTGTAATA	802

Oligonucleotides for reporter plasmid construction	Sequence	ID
Region i, sense	CTAGCAAAATAGGCTGTCCC	420
Region I, antisense	GGAGGATCCTGTCATTATGTTAAGTTTTATGC	654
Region ii, sense	ATAGGTACCGCAACATCCTGACCAATTATC	655
Region ii, antisense	TACGGATCCTTCTTGCAGGTGTTGGGAGA	787
Region iii, sense	CTGGGTACCAAAGATGTAGTCATGTATTAATCA	786
Region iii, antisense	CTTTATGTTTTTGGCGTCTTCCA	333
