# Supplementary material for: Etv2 regulates enhancer chromatin status to initiate Shh expression in the limb bud
Source: Nat Commun. 2022 Jul 21;13:4221. doi: 10.1038/s41467-022-31848-6 (PMC9304341; doi:10.1038/s41467-022-31848-6)
Supplement: Supplementary file 2 — Reporting Summary [file 41467_2022_31848_MOESM2_ESM.pdf]

nature portfolio

Corresponding author(s): Garry, D.J., Kawakami, Y.

Last updated by author(s): May 3, 2022

Reporting Summary

Nature Portfolio wishes to improve the reproducibility of the work that we publish. This form provides structure for consistency and transparency in reporting. For further information on Nature Portfolio policies, see our Editorial Policies and the Editorial Policy Checklist.

Statistics

For all statistical analyses, confirm that the following items are present in the figure legend, table legend, main text, or Methods section.

n/a	Confirmed	
☐	☒	The exact sample size (*n*) for each experimental group/condition, given as a discrete number and unit of measurement
☐	☒	A statement on whether measurements were taken from distinct samples or whether the same sample was measured repeatedly
☐	☒	The statistical test(s) used AND whether they are one- or two-sided<br>*Only common tests should be described solely by name; describe more complex techniques in the Methods section.*
☐	☒	A description of all covariates tested
☐	☒	A description of any assumptions or corrections, such as tests of normality and adjustment for multiple comparisons
☐	☒	A full description of the statistical parameters including central tendency (e.g. means) or other basic estimates (e.g. regression coefficient) AND variation (e.g. standard deviation) or associated estimates of uncertainty (e.g. confidence intervals)
☐	☒	For null hypothesis testing, the test statistic (e.g. F, t, r) with confidence intervals, effect sizes, degrees of freedom and P value noted<br>*Give P values as exact values whenever suitable.*
☐	☒	For Bayesian analysis, information on the choice of priors and Markov chain Monte Carlo settings
☒	☐	For hierarchical and complex designs, identification of the appropriate level for tests and full reporting of outcomes
☒	☐	Estimates of effect sizes (e.g. Cohen's *d*, Pearson's *r*), indicating how they were calculated

Our web collection on statistics for biologists contains articles on many of the points above.

Software and code

Policy information about availability of computer code

Data collection	The analysis was performed by R (v4.1.0) and all the codes for reproducing the results can be found at the github site:<br>https://github.com/djglab/Etv2_limb_manuscript<br>The raw reads were mapped to mouse genome (mm10) using Bowtie2 (v2.2.4), with the parameters -X2000 and -m1.
Data analysis	The analysis was performed by R (ver4.1.0) . The detailed analysis and other tools were described in the Methods section.

For manuscripts utilizing custom algorithms or software that are central to the research but not yet described in published literature, software must be made available to editors and reviewers. We strongly encourage code deposition in a community repository (e.g. GitHub). See the Nature Portfolio guidelines for submitting code & software for further information.

Data

Policy information about availability of data

All manuscripts must include a data availability statement. This statement should provide the following information, where applicable:

- Accession codes, unique identifiers, or web links for publicly available datasets
- A description of any restrictions on data availability
- For clinical datasets or third party data, please ensure that the statement adheres to our policy

The ATAC-seq dataset that supports the findings of this study have been deposited in Geo Expression Omnibus (GEO) database with the accession number GSE192865. All data will be available upon request.

Field-specific reporting

Please select the one below that is the best fit for your research. If you are not sure, read the appropriate sections before making your selection.

☒ Life sciences ☐ Behavioural & social sciences ☐ Ecological, evolutionary & environmental sciences

For a reference copy of the document with all sections, see nature.com/documents/nr-reporting-summary-flat.pdf

Life sciences study design

All studies must disclose on these points even when the disclosure is negative.

Sample size	For immunohistochemical and hitological analyses involving animal samples, it is standard to examine three independent embryos for reproducibility. All of the studies were repeated with minimum of three biological replicates and indicated if more samples were analyzed.
Data exclusions	No data excluded
Replication	All attempts at replication for standard assays (i.e. FACS, qPCR, immunohistochemistry) were successful. These techniques were routinely performed at least in triplicate. Transfection studies were performed in quadruple and repeated at least three times, which produced essentially the same results.
Randomization	The experimental groups are defined based on the genotypes. Therefore, randomization was not relevant to this study. For all of the experiments at least three embryos from the same genotypes were analyzed.
Blinding	Invetigators were blinded whenever possible. Cell counting studies were double blinded: imaging and counting were done by separate investigators with samples labeled solely by sample numbers.

Reporting for specific materials, systems and methods

We require information from authors about some types of materials, experimental systems and methods used in many studies. Here, indicate whether each material, system or method listed is relevant to your study. If you are not sure if a list item applies to your research, read the appropriate section before selecting a response.

Materials & experimental systems

n/a	Involved in the study	
☐	☒	Antibodies
☐	☒	Eukaryotic cell lines
☒	☐	Palaeontology and archaeology
☐	☒	Animals and other organisms
☒	☐	Human research participants
☒	☐	Clinical data
☒	☐	Dual use research of concern

Methods

n/a	Involved in the study	
☐	☒	ChIP-seq
☒	☐	Flow cytometry
☒	☐	MRI-based neuroimaging

Antibodies

Antibodies used

Antibody dilutions and amounts used are provided in Supplementary Table 1 and reproduced below:

Anti Active Caspase-3 clone C92-605 (1:200) BD Biosciences BD Cat#559565, RRID:AB_397274
Anti Desmin (1:250 for immunohistochemistry) Novus Novus Cat# NB120-15200, RRID:AB_789575
Anti E47 (0.2microg/reaction for EMSA) Santa Cruz Cat# sc-763X, RRID:AB_631405
Anti Endomucin (1:400 for immunohistochemistry) Abcam Cat# ab106100, RRID:AB_10859306
Anti Etv2 (0.2microg/reaction for EMSA, 2microg/reaction for ChIP) Sana Cruz Cat# sc-164278, RRID:AB_2100842
Anti GFP (1:400 for immunohistochemistry) Abcam Cat# ab13970, RRID:AB_300798
Anti HA (1:200 for immunohistochemistry) Roche Cat# 11867423001, RRID:AB_390918
Anti Hand2 (0.2microg/reaction for EMSA) Santa Cruz Cat# sc-9411, RRID:AB_2115993
Anti HoxD13 (0.2microg/reaction for EMSA) Santa Cruz sc-46364X
Anti Pax3 (1:200 for immunohistochemistry) DSHB Cat# PAX3, RRID:AB_528426
Anti phospho histone H3 (1:200 for immunohistochemistry) Millipore Cat# 05-806, RRID:AB_310016
Anti Prrx1 (1:100 for immunohistochemistry) Abcam Ab211292
Anti Smooth Muscle Actin (1:800 for immunohistochemistry) Thermo MS-1B-90
Anti-SHH (1:200 for immunohistochemistry) Santa Cruz Cat# sc-9024, RRID:AB_2239216
Control mouse IgG (0.2microg/reaction for EMSA) Jackson Cat# 015-000-003, RRID:AB_2337188
Control goat IgG (0.2microg/reaction for EMSA, 2 g/reaction for ChIP) Jackson Cat# 005-000-003, RRID:AB_2336985
Control Rabbit IgG (0.2microg/reaction for EMSA) Jackson Cat# 011-000-003, RRID:AB_2337118

Tie2-PE (0.2microg per million cells for FACS) e-bioscience Cat# 12-5987-83, RRID:AB_466101
CD31-PE (0.2microg per million cells for FACS) Becton Dickinson Cat# 553373, RRID:AB_394819
CD45-PE (0.2microg per million cells for FACS) e-bioscience Cat# 12-0451-82, RRID:AB_465668
CD16/CD32 (0.5microg per million cells for FACS) Becton Dickinson Cat# 553141, RRID:AB_394656

Validation

Please see above. RRID and links to the websites for each antibody are provided.

Eukaryotic cell lines

Policy information about cell lines

Cell line source(s)

Mouse: NIH3T3 cells ATCC CRL-1658.

Authentication

NIH3T3 cells were purchased from ATCC and no independent authentication was performed.

Mycoplasma contamination

Cell line was tested for micoplasma contamination by standard laboratory screening. All cell lines tested negative for microplasma contamination.

Commonly misidentified lines
(See ICLAC register)

No commonly misidentified cell lines were used in this study.

Animals and other organisms

Policy information about studies involving animals; ARRIVE guidelines recommended for reporting animal research

Laboratory animals

Etv2 knockout Ferdous et al., 2009 N/A
Etv2flox/flox Shi et al., 2015 N/A
Etv2(ER71)-EYFP Rasmussen et al., 2011 N/A
Etv2(ER71)-Cre Rasmussen et al., 2011 N/A
Etv2(ER71)-CreERT2 This paper N/A
ROSA26-LacZ Freidrich 1 et al., 991 RRID:IMSR_JAX:003309
ROSA26-ZsGreen1 Madisen et al., 2010 RRID:IMSR_JAX:007906
ROSA26-rtTA-ires-EGFP Belteki et al., 2005 RRID:IMSR_JAX:005670
iHA-Etv2 This paper/ Behrens et al. 2014 N/A
HoxB6-Cre Lowe et al., 2000 RRID:IMSR_JAX:017981
Shh-EGFP-Cre Harfe et al., 2004 RRID:IMSR_JAX:005622

All animals (males and females) used for breeding were between 8 weeks and 6 months of age. Embryos were staged and used at indicated embryonic stages. Sex of the embryos were not determined.

Animals were kept at 12 hour dark/light cycle, ambient temperature and humidity.

Wild animals

The study did not involve wild animalls

Field-collected samples

The study did not involve samples collected from the field.

Ethics oversight

All of our studies have been reviewed and approved by the Institutional Animal Care and Use Committee at the University of Minnesota.

Note that full information on the approval of the study protocol must also be provided in the manuscript.

ChIP-seq

Data deposition

☒ Confirm that both raw and final processed data have been deposited in a public database such as GEO.

☒ Confirm that you have deposited or provided access to graph files (e.g. BED files) for the called peaks.

Data access links
May remain private before publication.

The GEO accession number of the ATAC-seq data produced in this manuscript is GSE192865.

Files in database submission

The raw fastq files and processed data can be accessed at
748_FL_GFP_pos_S38_R1_001.fastq
748_FL_GFP_pos_S38_R2_001.fastq
748_HL_GFP_pos_S40_R1_001.fastq
748_HL_GFP_pos_S40_R2_001.fastq
753_HL_GFP_pos_S49_R1_001.fastq
753_HL_GFP_pos_S49_R2_001.fastq
753_FL_GFP_pos_S47_R1_001.fastq
753_FL_GFP_pos_S47_R2_001.fastq
758_FL_GFP_pos_S51_R1_001.fastq
758_FL_GFP_pos_S51_R2_001.fastq

758_HL_GFP_pos_S53_R1_001.fastq
758_HL_GFP_pos_S53_R2_001.fastq
Etv2CKO_posteriorHL_902_S1_R1_001.fastq
Etv2CKO_posteriorHL_902_S1_R2_001.fastq
Etv2CKO_posteriorHL_904_S2_R1_001.fastq
Etv2CKO_posteriorHL_904_S2_R2_001.fastq
ATAC_95_S30_R1_001.fastq
ATAC_95_S30_R2_001.fastq
ATAC_105_anterior_S31_R1_001.fastq
ATAC_105_anterior_S31_R2_001.fastq
ATAC_105_GFP_S32_R1_001.fastq
ATAC_105_GFP_S32_R2_001.fastq
LimbWT_1_12-4-20_S12_R1_001.fastq.gz
LimbWT_1_12-4-20_S12_R2_001.fastq.gz
LimbWT_2_12-4-20_S13_R1_001.fastq.gz
LimbWT_2_12-4-20_S13_R2_001.fastq.gz
Etv2cKO_11-07-20_S8_R1_001.fastq.gz
Etv2cKO_11-07-20_S8_R2_001.fastq.gz
Etv2cKO_10-31-20_S7_R1_001.fastq.gz
Etv2cKO_10-31-20_S7_R2_001.fastq.gz
Etv2cKO_12-1-20_S10_R1_001.fastq.gz
Etv2cKO_12-1-20_S10_R2_001.fastq.gz
Etv2cKO_11-28-20_S9_R1_001.fastq.gz
Etv2cKO_11-28-20_S9_R2_001.fastq.gz
Etv2cKO_12-9-20_S11_R1_001.fastq.gz
Etv2cKO_12-9-20_S11_R2_001.fastq.gz
916-10A_S7_R1_001.fastq.gz
916-10A_S7_R2_001.fastq.gz
916-10P_S14_R1_001.fastq.gz
916-10P_S14_R2_001.fastq.gz
916-1A_S1_R1_001.fastq.gz
916-1A_S1_R2_001.fastq.gz
916-1P_S8_R1_001.fastq.gz
916-1P_S8_R2_001.fastq.gz
916-2A_S2_R1_001.fastq.gz
916-2A_S2_R2_001.fastq.gz
916-2P_S9_R1_001.fastq.gz
916-2P_S9_R2_001.fastq.gz
916-3A_S3_R1_001.fastq.gz
916-3A_S3_R2_001.fastq.gz
916-3P_S10_R1_001.fastq.gz
916-3P_S10_R2_001.fastq.gz
916-4A_S4_R1_001.fastq.gz
916-4A_S4_R2_001.fastq.gz
916-4P_S11_R1_001.fastq.gz
916-4P_S11_R2_001.fastq.gz
916-7A_S5_R1_001.fastq.gz
916-7A_S5_R2_001.fastq.gz
916-7P_S12_R1_001.fastq.gz
916-7P_S12_R2_001.fastq.gz
916-9A_S6_R1_001.fastq.gz
916-9A_S6_R2_001.fastq.gz
916-9P_S13_R1_001.fastq.gz
916-9P_S13_R2_001.fastq.gz

Genome browser session
(e.g. UCSC)

Genome browser session for the ATAC-seq data at the ZRS region
https://genome.ucsc.edu/s/ndsouza/ZRS_region
https://genome.ucsc.edu/s/ndsouza/LMBR1_promoter
https://genome.ucsc.edu/s/ndsouza/OCC_Lmbr1
https://genome.ucsc.edu/s/ndsouza/OCC_Lmbr1_Promoter

Methodology

Replicates

ATAC-seq data has 2 or more replicates to validate reproducibility

Sequencing depth

748_FL_GFP_pos_S38_R1_001.fastq
total reads: 54639054
mapped reads: 47680427
uniquely mapped reads: 30543807

748_FL_GFP_pos_S38_R2_001.fastq
total reads: 54639054
mapped reads: 47680427
uniquely mapped reads: 30543807

748_HL_GFP_pos_S40_R1_001.fastq
total reads: 57213338
mapped reads: 47888101
uniquely mapped reads: 33437316

748_HL_GFP_pos_S40_R2_001.fastq
total reads: 57213338
mapped reads: 47888101
uniquely mapped reads: 33437316

753_HL_GFP_pos_S49_R1_001.fastq
total reads: 74612192
mapped reads: 67612825
uniquely mapped reads: 45981900

753_HL_GFP_pos_S49_R2_001.fastq
total reads: 74612192
mapped reads: 67612825
uniquely mapped reads: 45981900

753_FL_GFP_pos_S47_R1_001.fastq
total reads: 67829176
mapped reads: 62286361
uniquely mapped reads: 41762762

753_FL_GFP_pos_S47_R2_001.fastq
total reads: 67829176
mapped reads: 62286361
uniquely mapped reads: 41762762

758_FL_GFP_pos_S51_R1_001.fastq
total reads: 40722378
mapped reads: 38810665
uniquely mapped reads: 26725787

758_FL_GFP_pos_S51_R2_001.fastq
total reads: 40722378
mapped reads: 38810665
uniquely mapped reads: 26725787

758_HL_GFP_pos_S53_R1_001.fastq
total reads: 46619852
mapped reads: 43302845
uniquely mapped reads: 30982345

758_HL_GFP_pos_S53_R2_001.fastq
total reads: 46619852
mapped reads: 43302845
uniquely mapped reads: 30982345

Etv2CKO_posteriorHL_902_S1_R1_001.fastq
total reads: 82189062
mapped reads: 68858048
uniquely mapped reads: 52079207

Etv2CKO_posteriorHL_902_S1_R2_001.fastq
total reads: 82189062
mapped reads: 68858048
uniquely mapped reads: 52079207

Etv2CKO_posteriorHL_904_S2_R1_001.fastq
total reads: 66808320
mapped reads: 59731377
uniquely mapped reads: 46519769

Etv2CKO_posteriorHL_904_S2_R2_001.fastq
total reads: 66808320
mapped reads: 59731377
uniquely mapped reads: 46519769

ATAC_95_S30_R1_001.fastq
total reads: 171844242
mapped reads: 156195249
uniquely mapped reads: 111593942

ATAC_95_S30_R2_001.fastq

total reads: 171844242
mapped reads: 156195249
uniquely mapped reads: 111593942

ATAC_105_anterior_S31_R1_001.fastq
total reads: 185476114
mapped reads: 170789420
uniquely mapped reads: 136580928

ATAC_105_anterior_S31_R2_001.fastq
total reads: 185476114
mapped reads: 170789420
uniquely mapped reads: 136580928

ATAC_105_GFP_S32_R1_001.fastq
total reads: 167791752
mapped reads: 151643071
uniquely mapped reads: 105191364

ATAC_105_GFP_S32_R2_001.fastq
total reads: 167791752
mapped reads: 151643071
uniquely mapped reads: 105191364

LimbWT_1_12-4-20_S12_R1_001.fastq.gz
total reads: 137566112
mapped reads: 127103841
uniquely mapped reads: 87492446

LimbWT_1_12-4-20_S12_R2_001.fastq.gz
total reads: 137566112
mapped reads: 127103841
uniquely mapped reads: 87492446

LimbWT_2_12-4-20_S13_R1_001.fastq.gz
total reads: 136227980
mapped reads: 125591860
uniquely mapped reads: 89075384

LimbWT_2_12-4-20_S13_R2_001.fastq.gz
total reads: 136227980
mapped reads: 125591860
uniquely mapped reads: 89075384

Etv2cKO_11-07-20_S8_R1_001.fastq.gz
total reads: 155141888
mapped reads: 141855951
uniquely mapped reads: 108181017

Etv2cKO_11-07-20_S8_R2_001.fastq.gz
total reads: 155141888
mapped reads: 141855951
uniquely mapped reads: 108181017

Etv2cKO_10-31-20_S7_R1_001.fastq.gz
total reads: 206321174
mapped reads: 181691486
uniquely mapped reads: 141280465

Etv2cKO_10-31-20_S7_R2_001.fastq.gz
total reads: 206321174
mapped reads: 181691486
uniquely mapped reads: 141280465

Etv2cKO_12-1-20_S10_R1_001.fastq.gz
total reads: 37206578
mapped reads: 36320633
uniquely mapped reads: 29810444

Etv2cKO_12-1-20_S10_R2_001.fastq.gz
total reads: 37206578
mapped reads: 36320633
uniquely mapped reads: 29810444

Etv2cKO_11-28-20_S9_R1_001.fastq.gz
total reads: 153097026

mapped reads: 138476727
uniquely mapped reads: 109352511

Etv2cKO_11-28-20_S9_R2_001.fastq.gz
total reads: 153097026
mapped reads: 138476727
uniquely mapped reads: 109352511

Etv2cKO_12-9-20_S11_R1_001.fastq.gz
total reads: 140915224
mapped reads: 129941796
uniquely mapped reads: 103831987

Etv2cKO_12-9-20_S11_R2_001.fastq.gz
total reads: 140915224
mapped reads: 129941796
uniquely mapped reads: 103831987

916-10A_S7_R1_001.fastq.gz
total reads: 82356704
mapped reads: 70347619
uniquely mapped reads: 45736769

916-10A_S7_R2_001.fastq.gz
total reads: 82356704
mapped reads: 70347619
uniquely mapped reads: 45736769

916-10P_S14_R1_001.fastq.gz
total reads: 81027162
mapped reads: 70783860
uniquely mapped reads: 46654179

916-10P_S14_R2_001.fastq.gz
total reads: 81027162
mapped reads: 70783860
uniquely mapped reads: 46654179

916-1A_S1_R1_001.fastq.gz
total reads: 70084586
mapped reads: 63272746
uniquely mapped reads: 42633404

916-1A_S1_R2_001.fastq.gz
total reads: 70084586
mapped reads: 63272746
uniquely mapped reads: 42633404

916-1P_S8_R1_001.fastq.gz
total reads: 91793802
mapped reads: 83217390
uniquely mapped reads: 55185724

916-1P_S8_R2_001.fastq.gz
total reads: 91793802
mapped reads: 83217390
uniquely mapped reads: 55185724

916-2A_S2_R1_001.fastq.gz
total reads: 76395272
mapped reads: 66059935
uniquely mapped reads: 46279666

916-2A_S2_R2_001.fastq.gz
total reads: 76395272
mapped reads: 66059935
uniquely mapped reads: 46279666

916-2P_S9_R1_001.fastq.gz
total reads: 78277788
mapped reads: 69945835
uniquely mapped reads: 47548853

916-2P_S9_R2_001.fastq.gz
total reads: 78277788
mapped reads: 69945835

uniquely mapped reads: 47548853

916-3A_S3_R1_001.fastq.gz
total reads: 96100986
mapped reads: 82808168
uniquely mapped reads: 56725333

916-3A_S3_R2_001.fastq.gz
total reads: 96100986
mapped reads: 82808168
uniquely mapped reads: 56725333

916-3P_S10_R1_001.fastq.gz
total reads: 88720682
mapped reads: 78217060
uniquely mapped reads: 53135757

916-3P_S10_R2_001.fastq.gz
total reads: 88720682
mapped reads: 78217060
uniquely mapped reads: 53135757

916-4A_S4_R1_001.fastq.gz
total reads: 91673492
mapped reads: 80554363
uniquely mapped reads: 54588524

916-4A_S4_R2_001.fastq.gz
total reads: 91673492
mapped reads: 80554363
uniquely mapped reads: 54588524

916-4P_S11_R1_001.fastq.gz
total reads: 96762116
mapped reads: 84433319
uniquely mapped reads: 58192466

916-4P_S11_R2_001.fastq.gz
total reads: 96762116
mapped reads: 84433319
uniquely mapped reads: 58192466

916-7A_S5_R1_001.fastq.gz
total reads: 71202096
mapped reads: 62975103
uniquely mapped reads: 42032710

916-7A_S5_R2_001.fastq.gz
total reads: 71202096
mapped reads: 62975103
uniquely mapped reads: 42032710

916-7P_S12_R1_001.fastq.gz
total reads: 70622010
mapped reads: 61374899
uniquely mapped reads: 40154120

916-7P_S12_R2_001.fastq.gz
total reads: 70622010
mapped reads: 61374899
uniquely mapped reads: 40154120

916-9A_S6_R1_001.fastq.gz
total reads: 73808616
mapped reads: 63876864
uniquely mapped reads: 41982709

916-9A_S6_R2_001.fastq.gz
total reads: 73808616
mapped reads: 63876864
uniquely mapped reads: 41982709

916-9P_S13_R1_001.fastq.gz
total reads: 76407294
mapped reads: 67347867
uniquely mapped reads: 45382700

916-9P_S13_R2_001.fastq.gz
total reads: 76407294
mapped reads: 67347867
uniquely mapped reads: 45382700

Antibodies	*Describe the antibodies used for the ChIP-seq experiments; as applicable, provide supplier name, catalog number, clone name, and lot number.*
Peak calling parameters	macs2 callpeak -t treatment.bam -c control.bam -f BAM -g mm10 -n base_name --qvalue 0.05 --shift -100 --extsize 200 --nomodel
Data quality	Data quality for ATAC-seq was assessed with QC metrics by using FastQC software.
Software	bowtie2[2.4.2], macs2[2.2.7.1], picard[2.23.8], bedGraphToBigWig[v377], R [4.1.0]
